# Importance of manganese uptake in uropathogenic *Escherichia coli* CFT073 during urinary tract infection

**DOI:** 10.1128/mbio.03395-25

**Published:** 2026-06-26

**Authors:** A. E. Frick-Cheng, O. O. Adesunloro, B. Cobine, H. Heithoff, S. N. Smith, H. L. T. Mobley, A. E. Shea

**Affiliations:** 1 Department of Microbiology and Immunology, University of Michigan Medical School, Ann Arbor, Michigan, USA; 2 Department of Microbiology and Immunology, University of South Alabama Medical School, Mobile, Alabama, USA; University of Maryland, College Park, Maryland, USA

**Keywords:** manganese transport, UPEC, UTI, iron transport

## Abstract

Urinary tract infections (UTIs) are the second most common bacterial infection, with the majority of cases caused by uropathogenic *Escherichia coli* (UPEC). To establish infection, UPEC requires iron and other transition metals to support essential metabolic processes. In response, the host limits access to these metals during infection, generating a nutrient-restricted environment that pathogens must overcome. We investigated the role of three iron transport systems, Sit, Feo, and Efe, in the prototypical UPEC strain CFT073 during UTI. In the murine model of infection, loss of Sit, but not Feo or Efe, resulted in a significant fitness defect in the urine. Because Sit can transport both iron and manganese, we hypothesized that its contribution to virulence extends beyond iron import. Consistent with this idea, deletion of *sitABCD* (Δ*sit*) or both *sitABCD* and an additional manganese transporter *mntH* (Δ*sit/*Δ*mntH*) impaired growth in metal-restricted minimal medium. Interestingly, *mntH* deletion increased *sitA* transcription, but the inverse was not observed, suggesting compensatory regulation under cationic-limiting conditions. All three mutants (Δ*sit*, Δ*mntH*, and Δ*sit/*Δ*mntH*) displayed significant sensitivity to hydrogen peroxide-induced killing, with the double mutant exhibiting the strongest defect. In the murine model of ascending UTI, the Δ*sit/*Δ*mntH* strain was severely attenuated compared to wild type in the urine, bladder, and kidneys. Collectively, these findings demonstrate that manganese uptake, mediated primarily by Sit and MntH, is critical for oxidative stress resistance and bacterial fitness during infection. This highlights the importance of manganese acquisition as a determinant of UPEC pathogenesis in the iron-limited urinary tract.

**IMPORTANCE:**

Urinary tract infections (UTIs) are one of the most common infectious diseases worldwide, affecting over 50% of women. The primary culprit of uncomplicated UTIs is uropathogenic *Escherichia coli* (UPEC). Antibiotics are the standard therapy for treating UTIs; however, UPEC is steadily accumulating antibiotic resistance, increasing multidrug resistance. This increasing resistance severely complicates the clinical management of UTIs and requires alternative treatments. This study demonstrates that manganese transporters confer a critical fitness benefit to UPEC during UTI. Manganese acquisition allows UPEC to resist host attack; thus, targeting manganese uptake may lead to new strategies to combat difficult-to-treat UTIs.

## INTRODUCTION

Uropathogenic *Escherichia coli* (UPEC) causes the majority of urinary tract infections (UTIs), which affect 150 million people worldwide each year (1). When left untreated, UTIs can become a significant health problem and greatly diminish quality of life. Although UTIs occur in both men and women, ~81% of cases are in women (2). It is estimated that 50%–60% of women will experience at least one UTI during their lifetime (3), and ~25% of these cases recur within six months (4). Antibiotics are used to treat UTIs; however, UPEC is becoming increasingly resistant to antibiotics, making treatment for UTIs challenging (4). Thus, the development of effective alternative therapeutics is essential.

UTIs begin when bacteria, which can reside as commensal residents of the gastrointestinal tract (5), contaminate the urethral opening. Upon introduction, bacteria ascend the urethra, colonize and inflame the bladder, often leading to symptomatic bladder infection, or cystitis (5). UPEC can then ascend to the kidneys and cause infection in the upper urinary tract, known as pyelonephritis (6). The transmission of UPEC from the gastrointestinal tract through the upper urinary tract requires colonization of a variety of host environments, each with differing availability of nutrients, including the metal cations vital for survival of the bacterial cell (7).

Bacteria rely on a variety of metalloenzymes used in many essential processes, such as DNA synthesis and the detoxification of reactive oxygen species (ROS) (8), and iron is often the cofactor used by these enzymes (9). Consequently, *E. coli* has a variety of transporters to import iron from the milieu, such as FeoABC, EfeUOB, and SitABCD (10, 11). Because there is very little free iron in mammalian hosts due to nutritional immunity (12), UPEC can also acquire iron from extracellular sequestered sources via siderophores (5, 7). Given the fierce competition for iron that occurs during infection, it is likely that both systems provide an advantage to UPEC *in vivo* (13).

There also arises an interesting paradox within bacterial iron acquisition, particularly in pathogenic strains. While iron is required for several essential biological functions, excess iron can also result in intracellular oxidative damage through Fenton chemistry (14, 15). This issue is exacerbated during infection since neutrophils are recruited to the bladder and use ROS to kill UPEC (16, 17). To combat these challenges, bacteria use superoxide dismutases (SODs), metalloenzymes that eliminate superoxide radicals and hydrogen peroxide (18). UPEC strains have up to three SODs, one using manganese as a cofactor (MnSOD), one using iron (FeSOD), and a third that uses copper or zinc (Cu/ZnSOD) (19). MnSOD is the only SOD that provides a fitness advantage in the *in vivo* mouse model (19). UPEC also encodes transporters to acquire manganese, such as SitABCD, which can import both manganese and iron (20). Another is MntH, an integral membrane protein that serves as a proton co-transporter, not only transporting manganese but also other transition metals, such as iron, cadmium, and zinc, although with much lower affinity (21).

Due to the importance of iron during infection, we initially sought to define how different iron transporters contribute to the pathogenesis of UPEC. We constructed mutant strains lacking either the Feo, Efe, or Sit transport systems, which are responsible for transporting ferrous iron (Fe²^+^) across the inner membrane into the cytoplasm of UPEC (8). Interestingly, it was only the loss of Sit that resulted in a reduction of bacterial fitness in the mouse model of UTI. As Sit can also transport manganese, we shifted focus to the importance of manganese transport and made mutant strains lacking either MntH or both MntH and Sit. We found that the manganese transporter mutants had increased sensitivity to ROS and observed that knocking out both Sit and MntH results in an even more severe defect in the mouse than either mutant on its own. These experiments allowed us to gain a deeper understanding of how UPEC fuels critical physiological and metabolic processes while causing infection in the host.

## RESULTS

### Low-affinity iron transporters are highly prevalent in uropathogenic *Escherichia coli*

**Fig 1:**
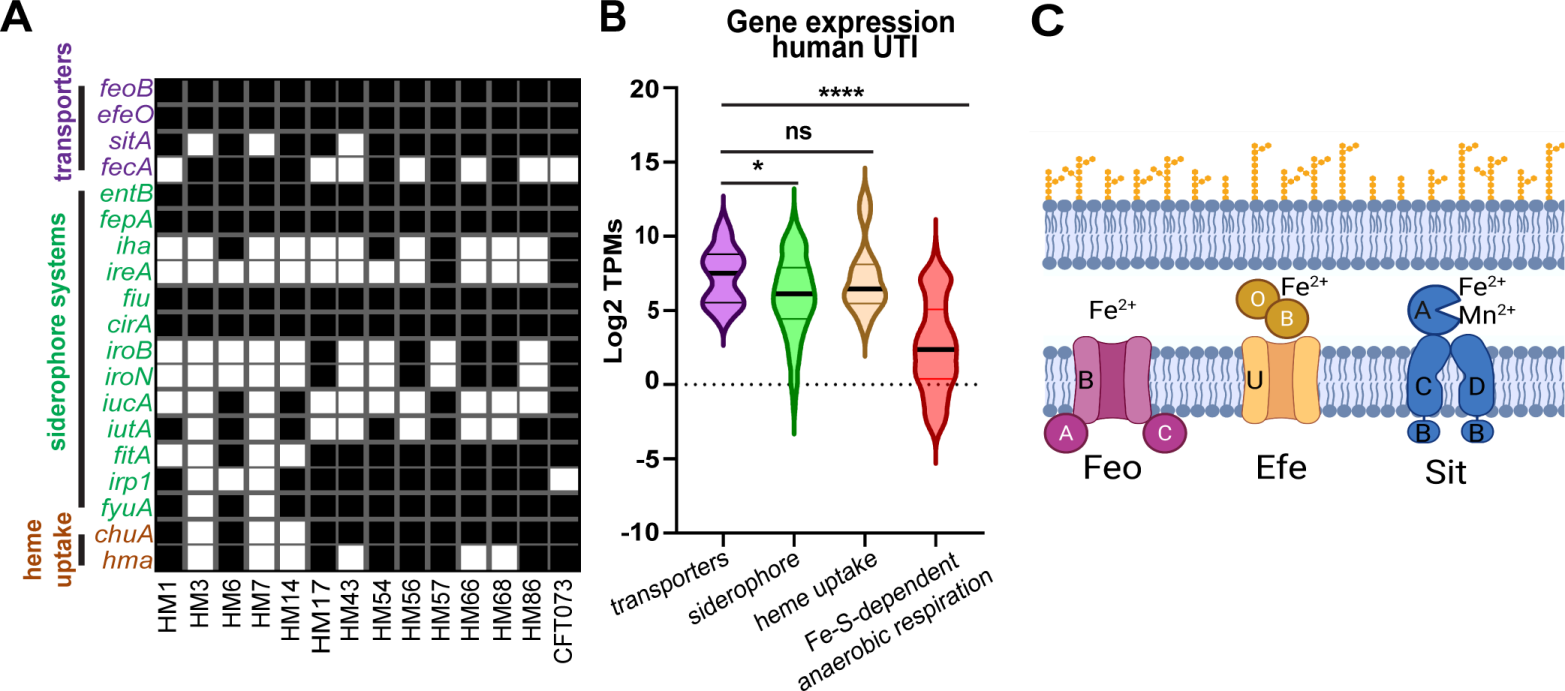
Inner membrane iron transporters are highly prevalent in uropathogenic *Escherichia coli*. (**A**) The presence or absence of iron acquisition and transport systems was assessed in a cohort of uropathogenic clinical isolates (HM) and prototypical UPEC type strain CFT073. White boxes indicate absence, and black boxes indicate the presence of the corresponding iron uptake system in each genome. (**B**) Gene expression of the corresponding iron acquisition and transport systems from the 14 clinical UPEC isolates was quantified from bacteria isolated from the urine of women with symptomatic UTI using normalized data, transcript per million (TPM). Data previously published in reference 24 but presented here with a unique analysis. Black lines indicate median with interquartile range, and statistical significance was assessed using the Kruskal-Wallis test with Dunn’s multiple comparison (*, 0.05; ****, *P* < 0.0001; ns, non-significant). (**C**) Schematic representation of iron transporters: FeoABC, EfeUOB, and SitABCD.

We analyzed the prevalence and sequence similarity of iron uptake systems (transporters, siderophores, and heme uptake) in a cohort of UPEC clinical isolates, comparing them to UPEC type strain CFT073 (Fig. 1A). All clinical isolates encoded the Feo and Efe iron transporters, as well as the siderophore enterobactin and its corresponding receptors, *fepA*, *fiu*, and *cirA* (Fig. 1A). We were unsurprised to see the consistent presence of enterobactin as it is associated with many diverse strains of *E. coli*, including non-pathogenic isolate K-12 (22). In contrast, other siderophore systems encoded by UPEC (aerobactin, yersiniabactin, and salmochelin), heme uptake systems, ferric citrate transport (*fecA*), and the Sit transporter were only sporadically present, with *sitA* in 10 of 13 strains (Fig. 1A). This less consistent presence does not mean these systems are dispensable for uropathogenesis. Indeed, several of these systems have been well characterized as virulence factors (23).

A previous study described the bacterial gene expression profiles of these specific clinical isolates during symptomatic UTI in women (24). This data set is a powerful tool to assess which genes are transcribed during active human infection and provides us with a snapshot into which systems UPEC is potentially utilizing to survive in the human host. During active infection, iron transporters (*feoABC*, *efeUOB*, and *sitABCD*) exhibited a significantly higher median expression level compared to Fe–S-dependent anaerobic respiration genes, which serve as a control group. Transporter expression was also significantly higher than the well-characterized siderophore systems, while remaining comparable to heme uptake systems (Fig. 1B). This suggests that these transport systems are highly utilized and may play important roles during human infection. This finding prompted us to investigate the roles of iron transporters in UPEC during UTI.

### Iron transport mutants display no observable *in vitro* growth defects

We constructed in-frame gene knockouts of ∆*feoABC* (∆*feo*), ∆*efeUOB* (∆*efe*), and ∆*sitABCD* (∆*sit*) inner membrane transporters (depicted in Fig. 1C) in prototype UPEC strain CFT073 to study their contributions to bacterial fitness. To test for generalized growth defects, we grew these mutant strains in LB medium and filter-sterilized pooled human urine *in vitro* and found none of the mutants had any growth defects compared to wild-type (WT) CFT073 (Fig. S1A and B). We also quantified iron chelation, indicative of siderophore production, via growth on the Chrome Azurol S (CAS) agar. We hypothesized that loss of these ubiquitous iron transport systems would lead to compensatory upregulation of siderophore production. However, we found that there was no significant difference between the mutants and WT (Fig. S1C).

### Sit, but not Feo or Efe, is a CFT073 fitness factor in the murine model of UTI

**Fig 2:**
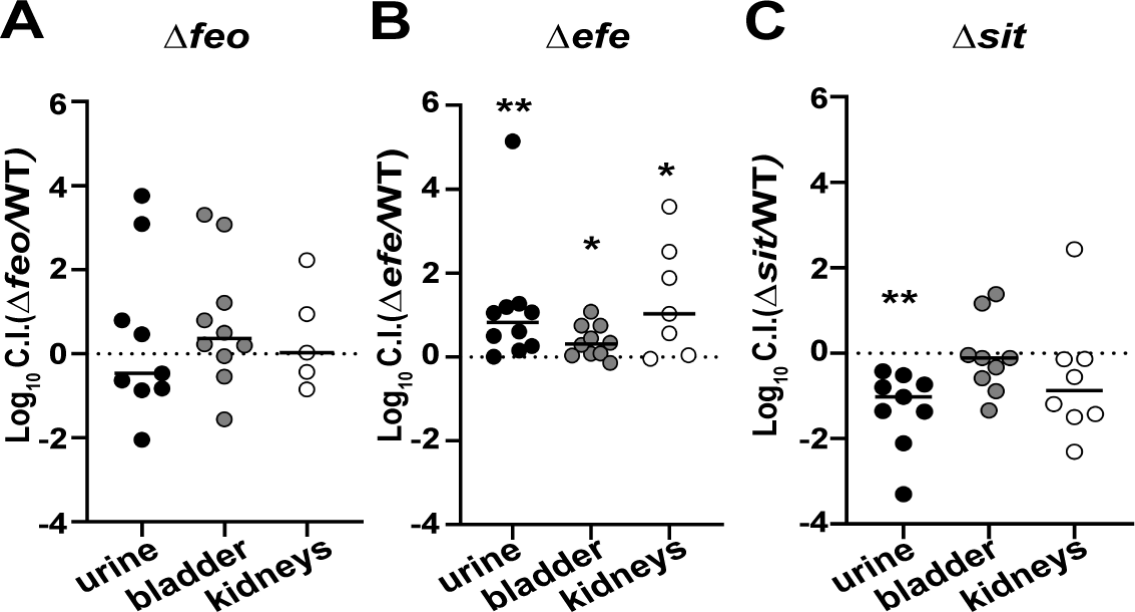
Sit contributes to UPEC strain CFT073 fitness during urinary tract infection. CBA/J mice were transurethrally co-infected for 48 h with CFT073 WT and isogenic mutants: (**A**) Δ*feo*, (**B**) Δ*efe*, and (**C**) Δ*sit*, each mixed at a 1:1 ratio with the wild type to generate a final inoculum of 10^8^ CFU/mL. Urine, bladder, and kidneys were collected at 48 h post-infection, and homogenates were plated on LB agar with and without selective antibiotics to quantify bacterial burdens of mutant and wild-type strains. Each data point represents the log_10_ C.I. from an individual mouse organ. Black lines indicate medians. The horizontal dashed line marks a log_10_ C.I. of 0, indicating equal fitness between strains; log_10_ C.I. >0 indicates higher fitness of the mutant, while log_10_ C.I. <0 indicates higher fitness of the wild type. Statistical significance was assessed with the Wilcoxon signed-rank test (*, *P* < 0.05; **, *P* < 0.01).

To assess the fitness of these mutants *in vivo,* we conducted three independent co-challenge experiments of WT against each isogenic iron uptake mutant in CBA/J mice (Fig. 2). Mice were transurethrally inoculated with 10^8^ colony-forming units (CFU) containing equal parts mutant and WT, and then urine, bladder, and kidneys were collected 48 h post-inoculation (Fig. 2; Fig. S2). Log_10_ competitive index (C.I.) >0 indicates the mutant strain has a fitness advantage over WT, and log_10_ C.I. <0 means WT has the advantage. The ∆*feo* mutant had no fitness defect compared to the WT (Fig. 2A), while the ∆*efe* mutant had a statistically significant increase in fitness in all three organ sites (Fig. 2B). The only mutant strain that was outcompeted by WT was ∆*sit*, which had a statistically significant defect (1-log, *P* = 0.0039) in the urine (Fig. 2C). Collectively, we concluded that among low-affinity iron uptake systems, the Sit transporter provides a fitness advantage to UPEC during UTI.

### *sitA* is significantly enriched in UPEC strains compared to environmental and fecal *E. coli* isolates

**Fig 3:**
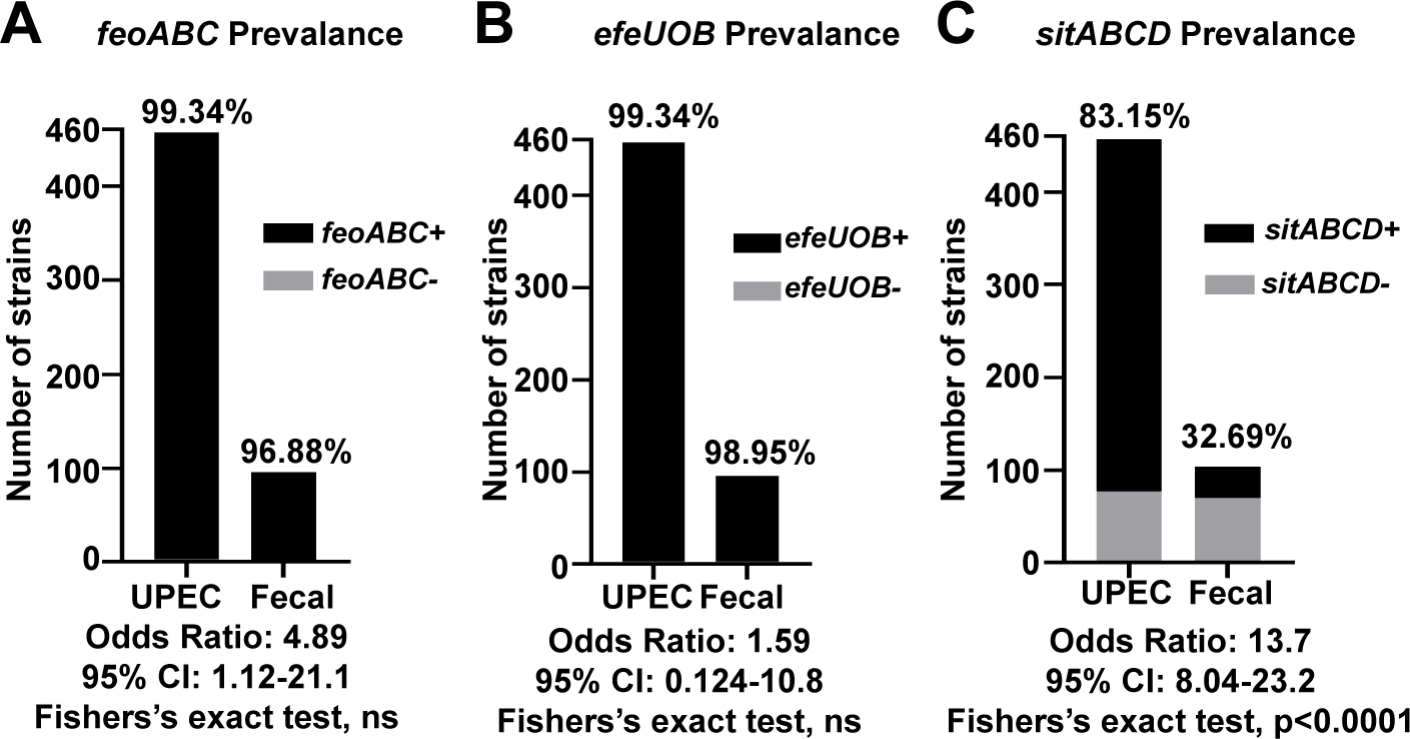
*sitABCD* is enriched in UPEC strains. The presence of (**A**) *feoABC*, (**B**) *efeUOB*, and (**C**) *sitABCD* was assessed in the genomes of 457 UPEC strains and 96 environmental or fecal *E. coli* strains. Strains encoding all of the tested genes are shown in black; those lacking any of the genes are shown in gray. Gene presence was defined by ≥80% protein identity and coverage. Statistical significance in gene enrichment between UPEC and fecal cohorts was assessed using Fisher’s exact test.

To understand why the Sit transporter provided a fitness advantage, rather than Feo or Efe transporters, we interrogated a cohort of publicly available sequenced genomes of both UPEC and environmental/fecal strains for the presence of these importers (Fig. 3). *feoABC* and *efeUOB* were nearly 100% conserved in both uropathogenic and environmental/fecal isolates (Fig. 3A and B), indicating that these systems are likely a hallmark of the *E. coli* species. Among the 457 UPEC genomes and 96 fecal *E. coli* genomes analyzed, *feoABC* was detected in 454 UPEC isolates (99.34%) and 93 fecal isolates (96.88%), while *efeUOB* was identified in 454 UPEC isolates (99.34%) and 95 fecal isolates (98.95%). In contrast, *sitABCD* was markedly enriched in UPEC isolates, being present in 380 of 457 UPEC isolates (83.15%) but only 34 of 104 fecal isolates (32.69%) (Fig. 3C), signifying the Sit transporter may be a UPEC-specific fitness factor.

**Fig 4:**
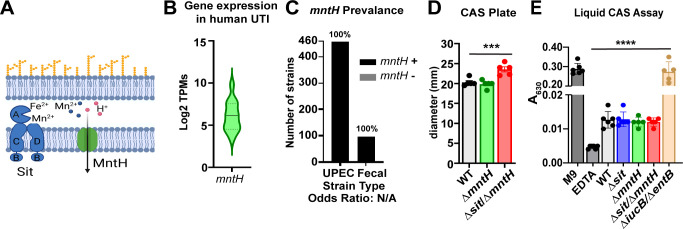
The Δ*sit*/Δ*mntH* double mutant has increased iron chelation activity on agar. (**A**) Schematic representation of inner membrane metal ion transporters: SitABCD and MntH. (**B**) Gene expression of mntH from the 14 clinical UPEC isolates was quantified from bacteria isolated from the urine of women with symptomatic UTI using normalized data, TPM. (**C**) The genomes of 457 UPEC strains and 96 environmental and fecal strains were examined for the presence of *mntH*. The number of strains containing *mntH* in their genome is indicated in black, and those without are indicated in gray. Fisher’s exact test indicated no significant difference in the odds ratio between the UPEC and fecal *E. coli* cohorts. (**D**) Siderophore production was assessed through growth on CAS agar. The mean of the diameter (mm) from independent trials (dots) is represented by bars, and the error bars represent the SEM. One-way ANOVA was performed to determine statistical differences (***, *P* < 0.001). (**E**) Strains grown in M9 minimal medium with 0.4% glucose showed no differences in iron chelation activity, as determined by the liquid CAS assay. Lower absorbance at 630 nm indicated greater chelation. EDTA and M9 medium served as positive and negative controls, respectively. Data represent the mean ± SEM of biological replicates, with significance determined by one-way ANOVA (****, *P* < 0.0001).

Besides iron, the Sit system can also import manganese (20). We hypothesized that manganese import, rather than iron import, is the cause of the Δ*sit* fitness defect observed *in vivo*. While iron is an essential cofactor for a myriad of enzymes, there are other functionally redundant enzymes that use manganese as the cofactor, specifically during iron limitation (25). Given that iron is severely limited during UTIs, UPEC might be relying more on manganese as a cofactor, and, therefore, manganese transporters would be critical during infection. To fully address this new hypothesis, we expanded our focus to include another manganese transporter, MntH (Fig. 4A) (26), and generated a *mntH* mutant (Δ*mntH*), as well as a double knock-out of both *sitABCD* and *mntH* (Δ*sit*/Δ*mntH*).

### Loss of both manganese transporters results in higher cumulative siderophore activity

We wanted to determine if *mntH*, like *sit*, is transcribed during active human infection and enriched in UPEC strains. We discovered that *mntH* had similar expression levels to *feoABC*, *efeUOB*, and *sitABCD* (Fig. 1B and 4B) during infection. However, MntH, like Feo and Efe, was 100% conserved in our representative cohorts of 457 UPEC isolates and 96 environmental/fecal isolated strains (Fig. 4C). Next, we wanted to ascertain if the single Δ*mntH* mutant or the Δ*sit/*Δ*mntH* double mutant had any differences in siderophore production. Interestingly, the Δ*sit/*Δ*mntH* mutant, unlike any of the single mutants shown in Fig. S1C, had significantly increased iron chelation activity on CAS agar (Fig. 4D), indicating that the double mutant compensates with higher siderophore activity. To obtain a more quantitative measure of siderophore production, we performed a liquid CAS assay using culture supernatants rather than halo diameter on CAS-containing agar. A CFT073 siderophore-deficient control strain lacking both aerobactin and enterobactin biosynthesis genes (Δ*iucB*/Δ*entB*) was included to validate assay specificity. Interestingly, under these conditions, we no longer observed the increased chelation activity of the Δ*sit/*Δ*mntH* mutant (Fig. 4E). However, CAS agar provides a cumulative measure of siderophore secretion over growth, whereas supernatant analysis reflects a single time point. Therefore, the double mutant may produce siderophores earlier or sustain higher production over time relative to the single mutants or WT.

### The Sit transporter aids UPEC utilization of manganese under iron limitation

**Fig 5:**
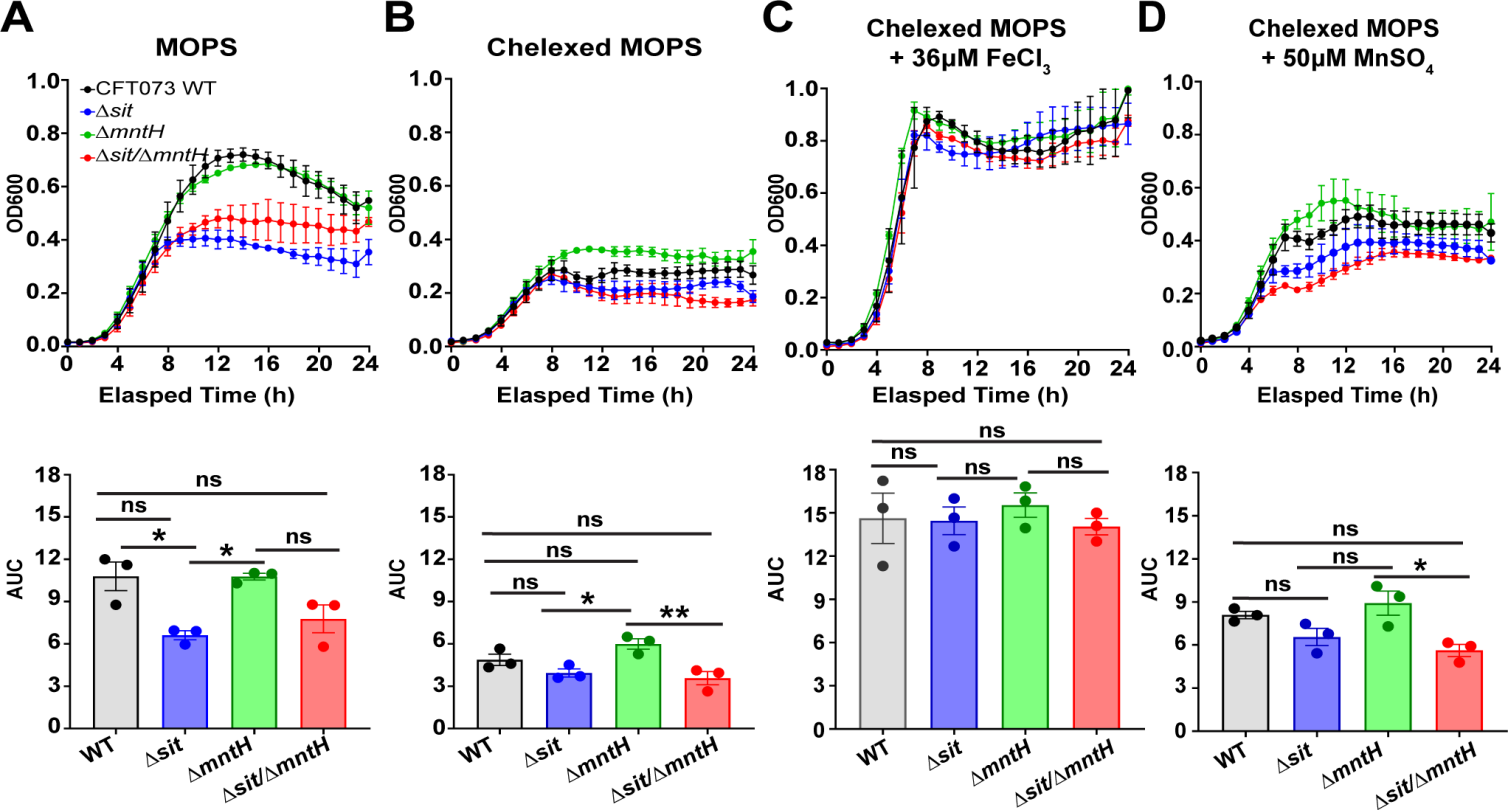
Loss of Sit attenuates UPEC growth in the presence of MnSO₄. WT and mutant strains (∆*sit*, ∆*mntH*, and Δ*sit/*Δ*mntH*) were grown in MOPS medium at 37°C with aeration under the following conditions: (**A**) MOPS without iron formulation, (**B**) chelex-treated MOPS (to remove iron and other cationic metals), (**C**) chelexed MOPS supplemented with 36 µM FeCl₃, and (**D**) chelexed MOPS supplemented with 50 µM MnSO_4_. All MOPS conditions were supplemented with 0.2% glucose. Optical density at 600 nm (OD_600_) was measured over 24 h, with data plotted hourly for clarity. Growth curves represent the mean of three biological replicates; error bars indicate ± SEM. Area under the curve (AUC) was calculated using GraphPad Prism. Each mutant was compared with the other mutants and the WT strain. Statistical significance was determined using ordinary one-way ANOVA with Tukey’s multiple comparisons test. Comparisons without marked significance were determined to show no significant difference (ns, non-significant; *, *P* < 0.05; **, *P* < 0.01).

To discern if loss of these transporters affects growth, we characterized the growth of mutant strains in an iron-limited, chemically defined medium. We performed growth curves of the mutants in morpholinopropane sulfonate (MOPS) medium (27) supplemented with 0.2% glucose, but without the traditional addition of FeSO_4_. Under these conditions, the Δ*sit* mutant and Δ*sit/*Δ*mntH* double mutant displayed the most severe growth defects compared to WT, but the Δ*mntH* strain was unaffected (Fig. 5A). We next grew the strains in MOPS medium that had been treated with Chelex-100, a chelating compound that removes polyvalent cations. Unsurprisingly, the growth defect was far more severe in all strains, though the trends observed in MOPS alone were recapitulated (Fig. 5B).

Because most trace metals are removed in chelexed MOPS, this medium can be supplemented with individual metals to assess mutant metal utilization under highly controlled conditions. Supplementation of chelexed MOPS with FeCl_3_ restored all mutants to WT growth levels, indicating no detectable defect in iron uptake under these conditions (Fig. 5C). This result is unsurprising since all the transport mutants still encode many other iron transport systems. However, when chelexed MOPS was supplemented with MnSO_4_, only Δ*sit/*Δ*mntH* had a subtle decrease in growth when compared to Δ*mntH* (Fig. 5D). Altogether, this indicates the Sit transporter might be more efficient than the MntH transporter at manganese import, or potentially that the Δ*mntH* single mutant is exhibiting a compensatory mechanism to boost its growth.

### Loss of *mntH* results in upregulation of *sitA*

To investigate potential genetic compensatory mechanisms in the mutant strains, we performed quantitative reverse transcription PCR (qRT-PCR) on WT and all three mutant strains. Gene expression of either *sitA* or *mntH* was assessed during growth in MOPS supplemented with glucose. As expected, there were undetectable levels of either *sitA* or *mntH* in either of their single mutants or the double mutant (Fig. S3). We did observe a significant twofold increase in *sitA* expression in the Δ*mntH* mutant (Fig. S3A). Conversely, a far more subtle, non-significant increase in *mntH* expression was observed in the Δ*sit* mutant (Fig. S3B). Taken together, we hypothesize that genetic compensation in the *mntH* mutant is contributing to the consistent robust growth of the Δ*mntH* mutant under metal-limited conditions (Fig. 5A and B). Furthermore, these results bolster the importance of the Sit transporter in growth under iron and manganese-limited conditions, such as MOPS, without iron formulation.

### Loss of manganese transport results in sensitivity to oxidative stress

**Fig 6:**
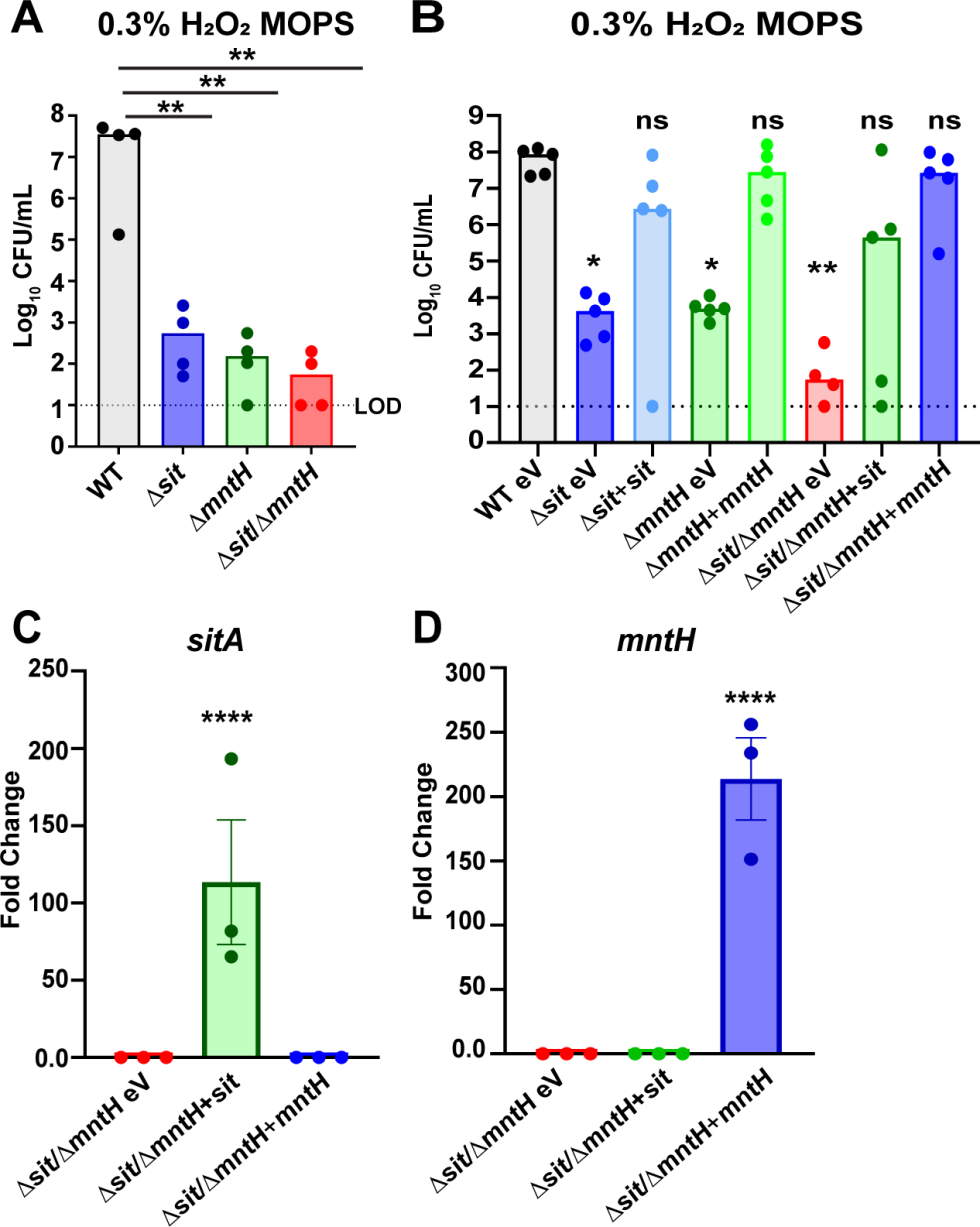
∆*sit*, ∆*mntH*, and ∆*sit/*∆*mntH* mutants exhibit increased sensitivity to oxidative stress. (**A**) WT and mutant strains (∆*sit*, ∆*mntH*, and ∆*sit/*∆*mntH*) were cultured in MOPS minimal medium supplemented with 0.3% hydrogen peroxide to induce ROS stress. Survival at 30 min is shown here, with the full-time course in Fig. S3. Bars are median, and individual dots are biological replicates. Statistical significance was assessed using the Kruskal-Wallis test with Dunn’s multiple correction to compare mutants to WT (**, *P* < 0.01). (**B**) Complementation of *sit* in ∆*sit* (∆*sit+sit*) and ∆*sit/*∆*mntH* (∆*sit/*∆*mntH+sit*) strains resulted in partial restoration of survival, while *mntH* complementation fully restored survival in both ∆*mntH* (∆*mntH+mntH*) and ∆*sit/*∆*mntH* (∆*sit/*∆*mntH+mntH*) strains. Empty vector (eV) plasmids were introduced into WT and mutant strains to control potential plasmid-related effects. Bars are median, and individual dots are biological replicates. Statistical significance was assessed using the Kruskal-Wallis test with Dunn’s multiple correction to compare complement strains and eV strains to the WT eV (*, *P* < 0.05; **, *P* < 0.01). Relative gene expression of (**C**) *sitA* and (**D**) *mntH* was measured in complemented strains in comparison to WT expressing empty vector (WT eV) using qRT-PCR and gapA as the reference gene. Strains were grown in LB for 24 h. Bars represent the mean of three biological replicates; individual data points are shown as dots. Statistical significance was assessed using a one-sample *t*-test (****, *P* < 0.0001).

Manganese may also contribute to bacterial virulence through its role in ROS detoxification. Indeed, UPEC relies on MnSOD to combat host responses during infection (19, 25, 28). Given this, we aimed to determine if the Δ*sit,* Δ*mntH*, or Δ*sit*/Δ*mntH* mutants had sensitivity to oxidative stress by incubating all strains in MOPS medium supplemented with 0.3% hydrogen peroxide (H_2_O_2_) for 1 h, enumerating CFU every 15 min (Fig. S4). All three mutants had a significant defect, which was most profound at the 30-min time point where the double mutant had a 6-log defect compared to the WT (Fig. 6A). These defects were able to be genetically complemented in *trans* (Fig. 6B). Interestingly, the addition of *mntH* alone (Δ*sit*/Δ*mntH+mntH*) was able to fully rescue the double mutant phenotype, while *sit* alone (Δ*sit/*Δ*mntH+sit*) resulted in only a partial rescue (Fig. 6B). Given the genetic compensation we discovered in the Δ*mntH* mutant (Fig. S3A), we performed qRT-PCR on the complemented strains to see if a similar phenomenon was occurring. Indeed, we observed a massive, 213-fold upregulation of *mntH* in the Δ*sit*/Δ*mntH+mntH* complemented strain, with a lesser, 113-fold upregulation of *sit* in the Δ*sit*/Δ*mntH+sit* complemented strain (Fig. 6C and D). Interestingly, the level of overexpression also correlates with the difference in complementation levels (Fig. 6B).

To evaluate how these transporters contribute to ROS detoxification under iron-limited conditions, we used the ROS-Glo assay to measure relative H_2_O_2_ levels in WT and mutant strains grown under normal (LB) and iron-depleted medium conditions (LB supplemented with iron-chelator, 2,2-dipyridyl [DIP]). In this assay, the medium alone has the highest level of H_2_O_2_, with the addition of bacterial strains reducing H_2_O_2_ due to their various detoxifying systems. As expected, all bacterial strains were able to detoxify H_2_O_2_ when compared to the medium alone in both LB and iron-depleted (LB+DIP) conditions (Fig. S5D). Interestingly, we observed that the Δ*sit* mutant had significantly less H_2_O_2_ in the media compared to the WT or any of the other mutant strains (Fig. S5D). Additionally, there was no difference in the Δ*sit* medium H_2_O_2_ levels between LB and iron-depleted, meaning this phenotype was independent of iron. We were very surprised by this result, given the sensitivity of this strain to exogenous H_2_O_2_ killing (Fig. 6A).

Therefore, we performed qRT-PCR of the Δ*sit* mutant grown in LB to assess expression of a select gene panel and determine whether compensation was specific to ROS detoxification or reflected broader metabolic reprogramming. Surprisingly, compared to WT, the Δ*sit* mutant showed downregulation of *katE* (catalase), *sodB* (FeSOD), and *sodC* (CuZnSOD), but no upregulation of ROS detoxification systems (Fig. S6). However, Δ*sit* exhibited increased expression of *fumB* with corresponding downregulation of *fumA* and *fumC.* Although these genes encode functionally redundant fumarases, FumB functions primarily in the reductive TCA pathway, suggesting a shift away from oxidative TCA metabolism toward a more reductive or anaerobic-like metabolic state in the Δ*sit* strain. Together, these findings suggest that loss of Sit induces broader metabolic reprogramming.

Interestingly, in standard LB, the WT and double mutant were able to reduce the amount of peroxide in the media. However, in the presence of the iron-chelator (DIP), the double mutant was significantly impaired in reducing the basal H_2_O_2_ compared to the WT (Fig. S5D). This is not due to a generalized growth defect, as both WT and the Δ*sit/*Δ*mntH* double mutant grow to the same levels in these conditions (Fig. S5A through C). In the iron-depleted environment, the bacteria presumably cannot use FeSOD and instead will have to utilize MnSOD to detoxify ROS. Given that the Δ*sit/*Δ*mntH* double mutant is missing both major manganese transporters, it could mean their intracellular manganese levels are low and now have only one enzyme (Cu/ZnSOD) to use. These results collectively indicate that the ability to transport manganese drastically affects UPEC survival and metabolism in the presence of ROS.

### Deletion of both Sit and MntH has a greater effect on fitness in the murine model of UTI

**Fig 7:**
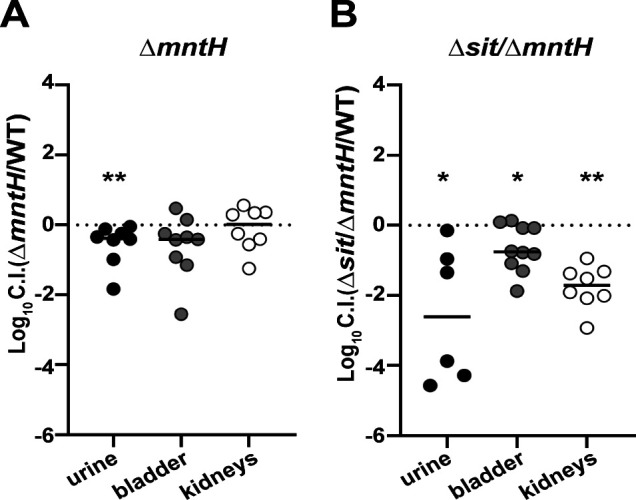
Loss of both Sit and MntH significantly reduces fitness during infection *in vivo*. CBA/J mice were transurethrally co-infected for 48 h with WT and isogenic mutants: (**A**) Δ*mntH* or (**B**) Δ*sit/*Δ*mntH*, mixed at a 1:1 ratio to generate a final inoculum of 10⁸ CFU/mL. Urine, bladder, and kidneys were collected at 48 h post-infection, and tissue homogenates were plated on LB agar with and without selective antibiotics to determine the bacterial burden of mutant and WT strains. Each data point represents the log_10_ C.I. for an individual mouse organ. Black lines indicate medians. The horizontal dashed line represents a log_10_ C.I. of 0, indicating equal fitness between WT and mutant strains. A log_10_ C.I. >0 indicates the mutant is more fit; a log_10_ C.I. <0 indicates the WT is more fit. Statistical significance was assessed with the Wilcoxon signed-rank test (*, *P* < 0.05; **, *P* < 0.01).

To confirm that manganese is important for UPEC during pathogenesis, we conducted *in vivo* co-challenge experiments with manganese-uptake mutants ∆*mntH* and ∆*sit*/Δ*mntH*. We expected the double mutant to display a greater defect against the WT than either of the single mutants, given the elimination of manganese uptake functional redundancy. These findings were confirmed in our murine model, where ∆*mntH* only had a significant fitness defect in the urine (Fig. 7A; Fig. S7), while the ∆*sit*/∆*mntH* mutant displayed significant defects in all three organ sites (Fig. 7B). While simultaneous loss of Sit and MntH yielded a significant fitness defect in both the bladder and kidney (Fig. 7B), neither single mutant was attenuated in these tissues (Fig. 2C and 7A).

**Fig 8:**
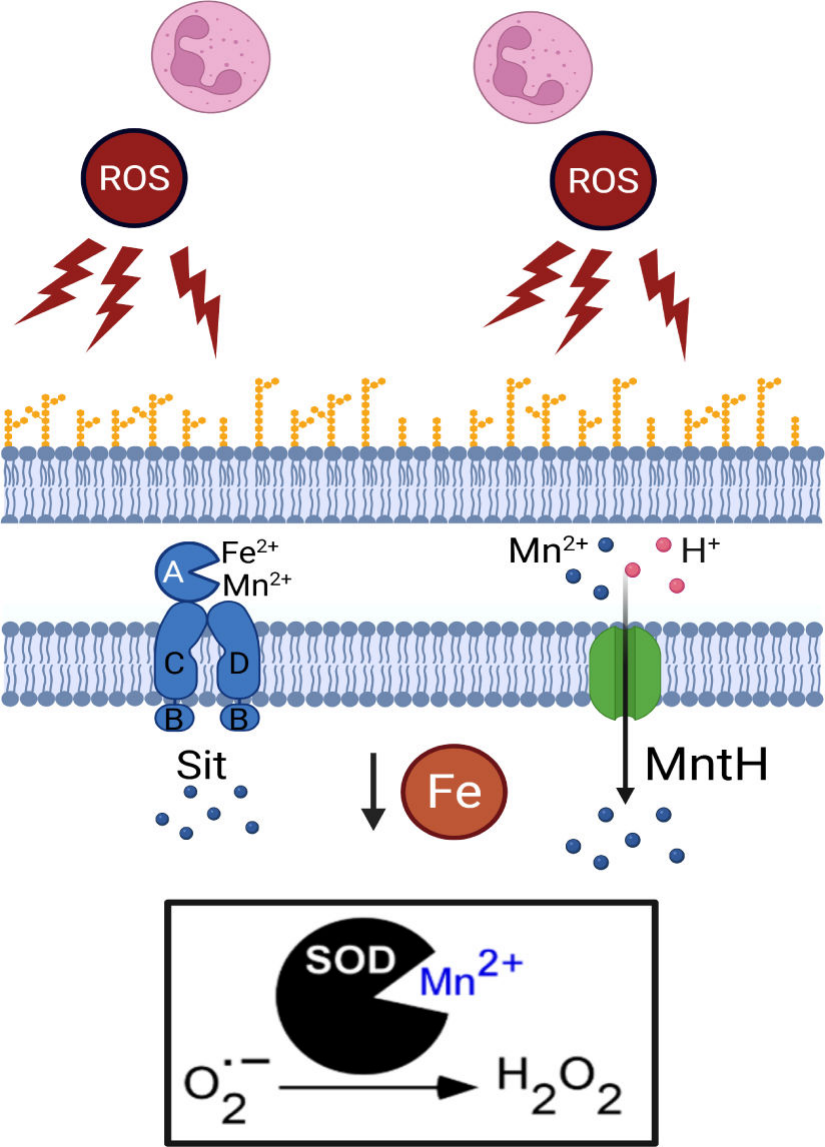
Proposed model illustrating the critical role of manganese transport in essential UPEC cellular processes required for survival in the urinary tract. This model represents our hypothesis that manganese functions as a vital cofactor for key bacterial cellular processes and is imported via the Sit and MntH transporters. This role becomes particularly important under iron-limited conditions, as observed in the urinary tract.

Since the manganese uptake mutants, particularly the double mutant, exhibited fitness defects compared to the WT, we conclude that the manganese transport systems, SitABCD and MntH, are critical for the fitness of UPEC during UTI. This likely reflects the essential role of manganese in protecting against hydrogen peroxide-induced oxidative stress within the iron-limited host environment (Fig. 8).

## DISCUSSION

The urinary tract is a highly iron-limited environment, and iron acquisition is critical for the survival and pathogenesis of UPEC, as iron is a vital cofactor involved in many catalytic, redox, and regulatory enzymatic reactions within the cell (29, 30). In response to iron limitation, UPEC has developed systems to scavenge iron, such as siderophores and heme receptors (31). While iron uptake during UTI is relatively well studied, much of this work is largely focused on siderophores or heme receptors (30). In this study, we initially focused on free iron transporters (Feo, Efe, and Sit). However, after finding that only the Sit transporter conferred an *in vivo* fitness advantage, we shifted our focus toward manganese transport, as the major distinction between Sit and the other two transporters is the ability of Sit to also import manganese. While iron is a vital cofactor for many essential biological processes (32), many environments, particularly the human host, are iron-limited (33). Consequently, bacteria have developed functionally redundant alternative enzymes that either do not use iron (34) or use a different metallic cation as a cofactor, which is often manganese (15). Therefore, we reasoned that UPEC might be relying on manganese to survive under iron limitation such as in the human host. Indeed, these results are consistent with findings in other pathogenic *E. coli* strains, such as avian pathogenic *E. coli* (APEC), where Sit contributes to bacterial fitness by mediating iron and manganese uptake (20).

To that end, we focused on other manganese transporters that UPEC might encode. The primary method of manganese uptake into the bacterial cell during infection is thought to be through the manganese transporter MntH (35) rather than Sit. Therefore, to fully study manganese transport, we made two additional mutants: a single Δ*mntH* mutant and a double Δ*sit*/Δ*mntH* mutant alongside the Δ*sit* mutant. The loss of Sit or Sit and MntH resulted in a strain with a growth defect in iron-limited minimal medium supplemented with manganese, indicating a reduction in manganese utilization, though this difference was only significant when compared to the Δ*mntH* strain and not the WT (Fig. 5D). We believe this is due to genetic compensation in the Δ*mntH* strain (Fig. 6A), where modest *sitA* upregulation likely provides a mild growth advantage. This effect is observed in cation-limited medium and manganese-supplemented iron-limited medium, thereby enhancing the differences between mutants. These results suggest that indeed the Sit transporter is important for manganese transport. It is interesting that we did not observe a corresponding genetic compensation of the MntH transporter in the Δ*sit* mutant. Perhaps UPEC relies more on the Sit transporter under iron-limited conditions rather than MntH. More studies are needed to understand the genetic interplay between these two transporters under iron limitation.

Flexible metabolism is a vital process that underpins the ability of UPEC to survive in the urinary tract (36). Particularly, the metabolic switch from anaerobic conditions in the gut to microaerobic conditions in the bladder (37). Furthermore, this essential process for UPEC survival in the urinary tract is reliant on *pckA*, an enzyme with predicted manganese binding sites (38). It is interesting to speculate that UPEC strains with reduced manganese uptake would display subtle metabolic defects in the iron-starved host that would lead to a loss of fitness. Interestingly, we found that the Δ*sit* mutant appeared to have different metabolic programming with an upregulation of *fumB*, a gene normally associated with anaerobic metabolism (Fig. S6). Future studies measuring metabolic output, such as lactate production, will allow us to more fully understand the metabolic dysregulation occurring in manganese uptake mutants under iron limitation.

Neutrophils are a major part of the initial immune response against UPEC (28). These immune cells use ROS to eliminate bacterial invaders, and, in response, UPEC encodes three SOD enzymes to detoxify ROS, relying most heavily on the SOD that utilizes manganese as a cofactor (19). Therefore, we hypothesized that loss of manganese transporters would result in increased sensitivity to exogenous ROS, as was observed in APEC (20). Both ∆*sit* and ∆*mntH* mutants were highly sensitive to ROS-mediated killing compared to the WT, and the double ∆*sit/*∆*mntH* mutant was even more sensitive (Fig. 6). This result could explain the loss of *in vivo* fitness we observed in the Δ*sit* mutant, as, presumably, these cells would be unable to withstand the oxidative burst neutrophils employed. Additionally, this sensitivity might be even further exacerbated by another protein that neutrophils use, calprotectin, which comprises 50% of the protein content in the neutrophil cytoplasm and sequesters both manganese and zinc (39). Manganese is not otherwise sequestered by the host (40). It would be interesting in future studies to explore the susceptibility of these mutant strains to being killed by neutrophils or determine if these mutants no longer have a defect in a neutropenic murine model.

Given the sensitivity to ROS observed in the ∆*mntH* and the ∆*sit/*∆*mntH* double mutant, we wanted to see if these mutants also had *in vivo* fitness defects. Therefore, we performed a second co-challenge experiment comparing the single ∆*mntH* mutant or the double ∆*sit/*Δ*mntH* mutant against WT (Fig. 7). Like the ∆*sit* mutant, loss of Δ*mntH* was not enough to cause a loss of fitness beyond an incredibly subtle defect in urine. This is interesting since the Δ*mntH* mutant was just as sensitive as the Δ*sit* mutant in the H_2_O_2_ assays. However, it seems that *in vivo,* the redundancy provided by Sit is sufficient for its survival. The double mutant had strongly significant defects in all three organ sites when compared with WT, which were larger than either single mutant. We believe this is due to eliminating the redundancy of manganese importers, as has been documented with other classes of transporters (41).

In summary, our findings demonstrate that manganese importers are critical determinants of UPEC fitness and virulence during UTI. *In vivo* attenuation observed with the Δ*sit* and Δ*sit*/Δ*mntH* mutants indicates that Sit and MntH represent the major physiologically relevant systems used by UPEC during infection. Our data confirm the critical role manganese plays in ROS survival and growth during iron-limited conditions. These collective biological and physiological findings identify bacterial manganese acquisition as a potential therapeutic vulnerability for UTIs and possibly other infections where pathogens encounter host-derived oxidative stress and iron limitation.

## MATERIALS AND METHODS

### Bacterial strains, growth conditions, mutant construction, and genetic complementation

UPEC type strain CFT073 was grown at 37°C under aerated conditions in LB, MOPS, M9, and filter-sterilized human urine. Mutants and complemented strains were grown with appropriate antibiotics. Growth curves were performed in a Bioscreen-C automated growth curve machine (Growth Curves USA). Mutants were constructed using the lambda red recombinase system (42), and complementation plasmids were constructed using Gibson assembly. Mutants and complemented strains are detailed in Table 1, and primers used for mutagenesis and cloning are in Table 2. See Supplemental Methods for detailed descriptions.

**TABLE 1:** List of strains and plasmids

Strain or plasmid	Genotype or description	Reference or source
Strains		
CFT073 (WT)	*E. coli* strain isolated from a patient with acute pyelonephritis	(43)
Δ*efe*	CFT073 *efeOUB*::spec, Spec^r^	This study
Δ*feo*	CFT073 *feoABC*::cam, Cam^r^	This study
Δ*sit*	CFT073 *sitABCD*::kan, Kan^r^	This study
Δ*mntH*	CFT073 *mntH*::cam, Cam^r^	This study
Δ*sit/mntH*	CFT073 *sitABCD*::kan *mntH*::cam, Kan^r^, Cam^r^	This study
WT eV	CFT073 expressing empty pGEN vector, Amp^r^	This study
Δ*sit* eV	Δ*sit* expressing empty pGEN vector, Amp^r^	This study
Δ*mntH* eV	Δ*mntH* expressing empty pGEN vector, Amp^r^	This study
Δ*sit/mntH* eV	Δ*sit/mntH* expressing empty pGEN vector, Amp^r^	This study
Δ*sit^+sit^*	Δ*sit* expressing *sit* in *trans* under the control of the native promoter, Amp^r^	This study
Δ*mntH^+mntH^*	Δ*mntH* expressing *mntH* in *trans* under the control of the native promoter, Amp^r^	This study
Δ*sit/*Δ*mntH^+mntH^*	Δ*sit/mntH* expressing *mntH* in *trans* under the control of the native promoter, Amp^r^	This study
Δ*sit^+sit^*/Δ*mntH*	Δ*sit/mntH* expressing *sit* in *trans* under the control of the native promoter, Amp^r^	This study
Plasmids		
pGEN eV	Low copy number, promoterless plasmid, Amp^r^	(44)
pGEN *sit*	*sitABCD* with native promoter, cloned from CFT073 via Gibson assembly, Amp^r^	This study
pGEN *mntH*	*mntH* with native promoter, cloned from CFT073 via Gibson assembly, Amp^r^	This study

**TABLE 2:** Primers used in the study^*e*^

Gene or plasmid	Forward primer	Reverse primer
*efe^a^*	TCTCATTATATTGCGCGAAGGACTTG AAGCCGCGCTGATTGTCAGTTTGA GTGTAGGCTGGAGCTGCTTC	CAGTGTACGAACGCCTGGT GCGGCGTAAACGCCTTATC CGCCTAAAAACAATGGGAAT TAGCCATGGTCC
*feo^a^*	AAACGCCCCATGAGGTAGTTATCCAG TTAATGAGAAACAAGTAGGCACCT GTGTAGGCTGGAGCTGCTTC	GCCAGGATGCAAACGCATA CCGGGCTTTCAAAACAGGC AGTGAAACAAGGATGGGAAT TAGCCATGGTCC
*sit^a^*	TTTGTTCTAATTTTGCTCACTATAGGT ACTAAATTGTGTAGGCTGGAGCTGCT TC	AAAAGTATCGGGTGTTGCTG TCCTCGGATGTTGTGATGGG AATTAGCCATGGGAATTAGC CATGGTCC
*mntH^a^*	ATCCGCCAATGGTGCCAAATGCGAC GCTCATTCAAATGGGAATTAGCCATG GTCCGTGTAGGCTGGAGCTGCTTC	GAAACATAGCAAAGGCTATG TTTTAGAGGCACAAGGTGTA GGCTGGAGCTGCTTCATGG GAATTAGCCATGGTCC
*efe^b^*	ATGATCATCGGTGCGCTGACG	TGACGAAGTTGCTACCGGAGG
*feo^b^*	ATCTGGCAATACACGCCTTGCC	CAGCCAACACAGCGAATAGGG
*sit^b^*	GAGAGGTGCAAATCCTCCCATAC	GCTGGATTGAAATGTTCGGGC
P_native_*sit^c^*	ATTCCTGCAGGGCATGCCCCCTAAT TGGGTGATAACAGATCCTGTGTATG	GTACCAAGCTTCATATGCCCTCTGTTT TCATAAAAGCAAAGTCAACAAGAT
P_native_*mntH^c^*	ATTCCTGCAGGGCATGCCCCCTACA ATCCCAGTGCCGTTCCTAC	GTACCAAGCTTCATATGCCCATTCTAT GTAACATGGTTAAGGAAAACGAGATC
pGEN^*c*^	GGGCATATGAAGCTTGGTACCG	GGGGCATGCCCTGCAGGAAT
*gapA^d^*	CGACCTGTTAGACGCTGATTAC	CGATCAGATGACCGTCTTTCAC
*sitA^d^*	GCGAGAGTACGATTTCCGATAA	GTGCTCAGGAATCGACATAGAG
*mntH^d^*	CCAGCTTCGGCTATCAGTTATT	CAGTTTGGCCGAGAGGATTT
*sodA^d^*	TAACCAGGACTCTCCACTGAT	ACGGCGGTTCTGGAATTT
*sodB^d^*	GTACCGCGTTTGAAGGTAAATC	CAGGCAGTTCCAGTAGAAAGTA
*sodC^d^*	CACAATGAACGGAGGTCCTAT	GACGAGGTTCATCTCGACTTT
*fumA^d^*	GCCTGTCCTCCGTATCATATTG	TCCGTTGGCAGTTCATCATAG
*fumB^d^*	CTGATTGACGCCGGTAAAGA	GCCAAGTGAACCTGATGGATA
*fumC^d^*	GCTGCGGAATTGGTGAAATC	TGACAGCAGAGCATGGTTAAT
*katG^d^*	GTGGTCATACGCTGGGTAAA	CCCAGCCTAAACCTTGTTCT
*katE^d^*	GGAAACCACTGGCAGGTAAA	TCAATCGCTTCCCACAACTC
*oxyR^d^*	GTGGCAATGGAGGATGGATAA	GCTAACGTGGCAGGAATCT

^*a*^ Used for lambda red mutagenesis.

^*b*^ Used for mutagenesis confirmation.

^*c*^ Used for Gibson assembly.

^*d*^ Used for qRT-PCR.

^*e*^ Primers are listed 5’ to 3’; underlined sequences for mutant construction indicate regions homologous to the gene of interest.

### *In silico* analysis

With the protein Basic Local Alignment Search Tool, we used the PATRIC database (45) to determine the amino acid percent identity of several iron acquisition proteins present in *E. coli* strains. For determining the prevalence of the *feoABC*, *efeUOB*, *sitABCD, and mntH* genes, 457 UPEC genomes and 96 fecal or environmental *E. coli* genomes were analyzed using a cohort in a previously published paper (23). The presence of these genes was determined by ≥80% protein sequence similarity and coverage.

### Murine model of ascending UTI co-challenge experiments

The individual fitness of each mutant was assessed in the CBA/J mouse model of ascending UTI (46). Each mutant was co-inoculated with WT in a 1:1 ratio into murine bladders. After 48 h of infection, urine was collected, and the bladders and kidneys were aseptically removed. CFU was enumerated in each organ site using plain and antibiotic-containing LB agar to differentiate between the WT and mutant. Counts were determined using QCount software (Spiral Biotech), and then the number of mutant CFU (antibiotic plates) was subtracted from the total number of CFU (plain plates) to determine the number of bacteria present in the input and output. The competitive index (C.I.) was calculated using the following formula:


$$\frac{\frac{mutant\;output}{WT\;output}}{\frac{mutant\;input}{WT\;input}}.$$

A log_10_ C.I. value less than 0 indicates that the WT strain outcompetes the mutant, whereas a log_10_ C.I. greater than 0 indicates that the mutant outcompetes the WT. See Supplemental Methods for a detailed description.

### CAS assay

To assess siderophore production, the CAS agar assay was performed as previously described (47, 48). Bacterial strains were cultured overnight at 37°C in LB. Then, 5 μL of each overnight culture was spotted onto CAS agar plates and incubated at 37°C for 18–20 h to allow for siderophore production and detection. A visible color change of agar from blue to orange around bacterial growth indicates siderophore production. The diameter (mm) of the zones was measured using a ruler and recorded. For the liquid CAS assay, the overnight cultures were washed once with PBS, diluted 1:100 into M9 minimal medium supplemented with 0.4% glucose, and incubated overnight, with aeration at 37°C. Cultures were pelleted by centrifugation, and 100 μL of supernatant was combined with 100 μL of CAS shuttle solution prepared according to an established protocol (49), with 10 mM EDTA used as a positive control (AmBeed catalog no. A354454-100g). Solutions were incubated at room temperature for 30 min, and the absorbance at 630 nm was recorded. See Supplemental Methods for a detailed description.

### Hydrogen peroxide assays

Overnight cultures of ∆*sit*, ∆*mntH*, and ∆*sit/*∆*mntH* were inoculated into LB medium overnight. The next day, cultures were normalized to ~10^8^ CFU/mL, then washed in MOPS with an iron-free formulation of nutrient mix and 0.2% glucose. All hydrogen peroxide assays were performed under static conditions at room temperature, and samples were collected at 15, 30, 45, and 60 min post-inoculation. Following incubation of LB agar plates at 37°C overnight, viable colonies were enumerated at countable dilutions.

### Extraction of RNA and qRT-PCR analysis

Strains were grown to mid-log, and cultures were then pelleted. The pelleted bacteria were resuspended in 500 μL of RNA Protect (Qiagen) and incubated at room temperature for 5 min. Samples were then pelleted, washed with RNase/DNase-free water, pelleted, and frozen at −80°C. RNA was extracted using the RNeasy minikit (Qiagen) after treatment with lysozyme and proteinase K. RNA samples were then DNase-treated using a Turbo DNA-free kit (Invitrogen). After confirming no DNA was present via PCR with gDNA primers, 1,000 ng of RNA were reverse transcribed into cDNA using iScript (Bio-Rad). qRT-PCR was performed on 5 ng of the template cDNA using a Lightcycler 480 II PCR machine (Roche) using SYBR green mastermix (FisherBrand). Samples were analyzed using the 2^−ΔΔCT^ method, with *gapA* as the housekeeping gene. Primers used are listed in Table 2.

### Statistics and software

All graphs and charts were plotted, and statistics were calculated using GraphPad Prism 10. To calculate the statistical difference in odds ratio, Fisher’s exact test was used. To calculate statistical significance for all bar graphs, one-way ANOVA with Tukey’s or Dunn’s multiple comparisons test was used. For mouse co-challenge experiments, statistical significance was calculated using the Wilcoxon signed-rank test. One one-sample *t*-test was used to assess statistical significance for the qRT-PCR data. *P < 0.05, **P < 0.01, ***P < 0.001, ****P < 0.0001.

## SUPPLEMENTAL MATERIAL

Supplemental Figures mbio.03395-25-s0001.pdf Figures S1 to S7.

Supplemental Methods mbio.03395-25-s0002.docx Additional method details.

ASM does not own the copyrights to Supplemental Material that may be linked to, or accessed through, an article. The authors have granted ASM a non-exclusive, world-wide license to publish the Supplemental Material files. Please contact the corresponding author directly for reuse.
