# Supplementary material for: Importance of manganese uptake in uropathogenic Escherichia coli CFT073 during urinary tract infection
Source: mBio. 2026 Jun 26;17(8):e03395-25. doi: 10.1128/mbio.03395-25 (PMC13463694; doi:10.1128/mbio.03395-25)
Supplement: Supplementary file 1 [file mbio.03395-25-s0001.pdf]

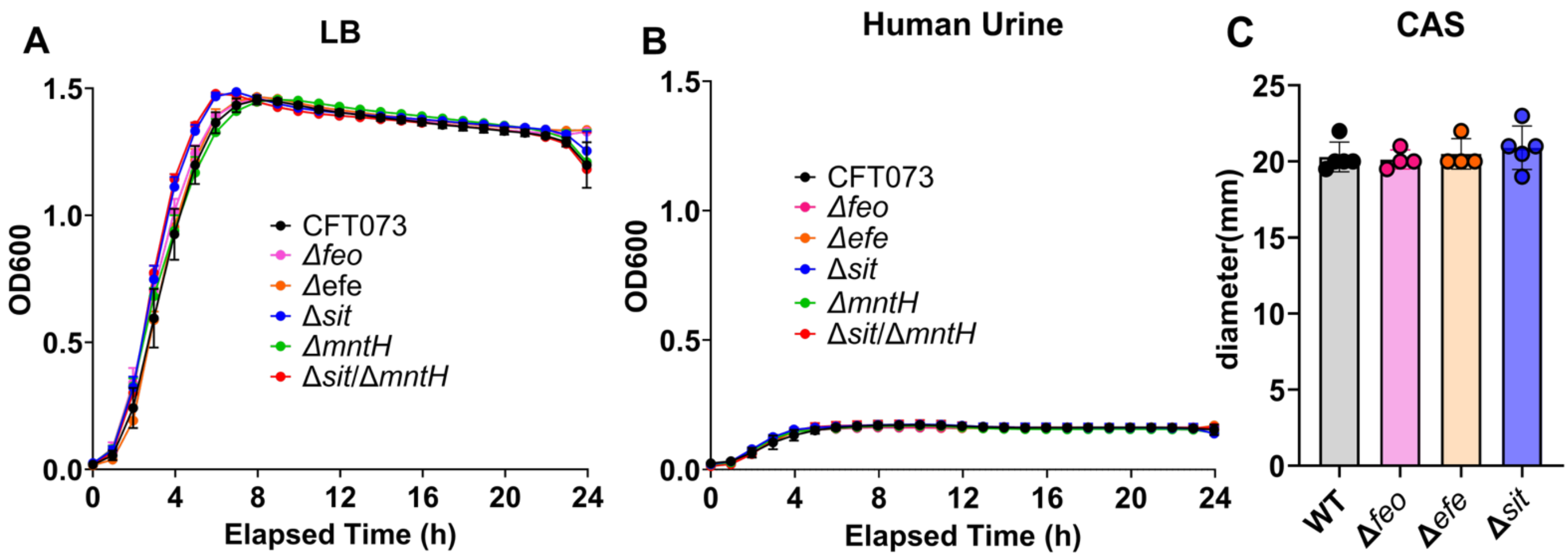

Figure S1. There are no observable growth defects *in vitro* with iron transport mutants ∆*feo*, ∆*efe*, ∆*sit*, ∆*mntH*, Δ*sit*/Δ*mntH*. Bacterial growth of wild type CFT073 and mutants ∆*feo*, ∆*efe*, ∆*sit*, ∆*mntH and* ∆*sit*/∆*mntH* was measured in (**A**) LB medium and (**B**) human urine grown at 37ºC with aeration. Average OD_{600} for each strain was recorded every 15 minutes for 24 h with data plotted hourly for clarity. Data shown are the averages from three to five biological replicates, and error bars represent standard error of the mean (SEM). **(C)** CFT073 wild type (WT), Δ*feo*, Δ*efe*, and Δ*sit* mutants were grown overnight at 37°C in LB, and 5µL of each culture was spotted onto CAS agar plates. Plates were incubated at 37°C for 20 hours to assess siderophore production. The diameter (mm) of the color change zone was measured. Each dot represents a biological replicate. Statistical analysis was assessed using one-way ANOVA; no significant differences were observed.

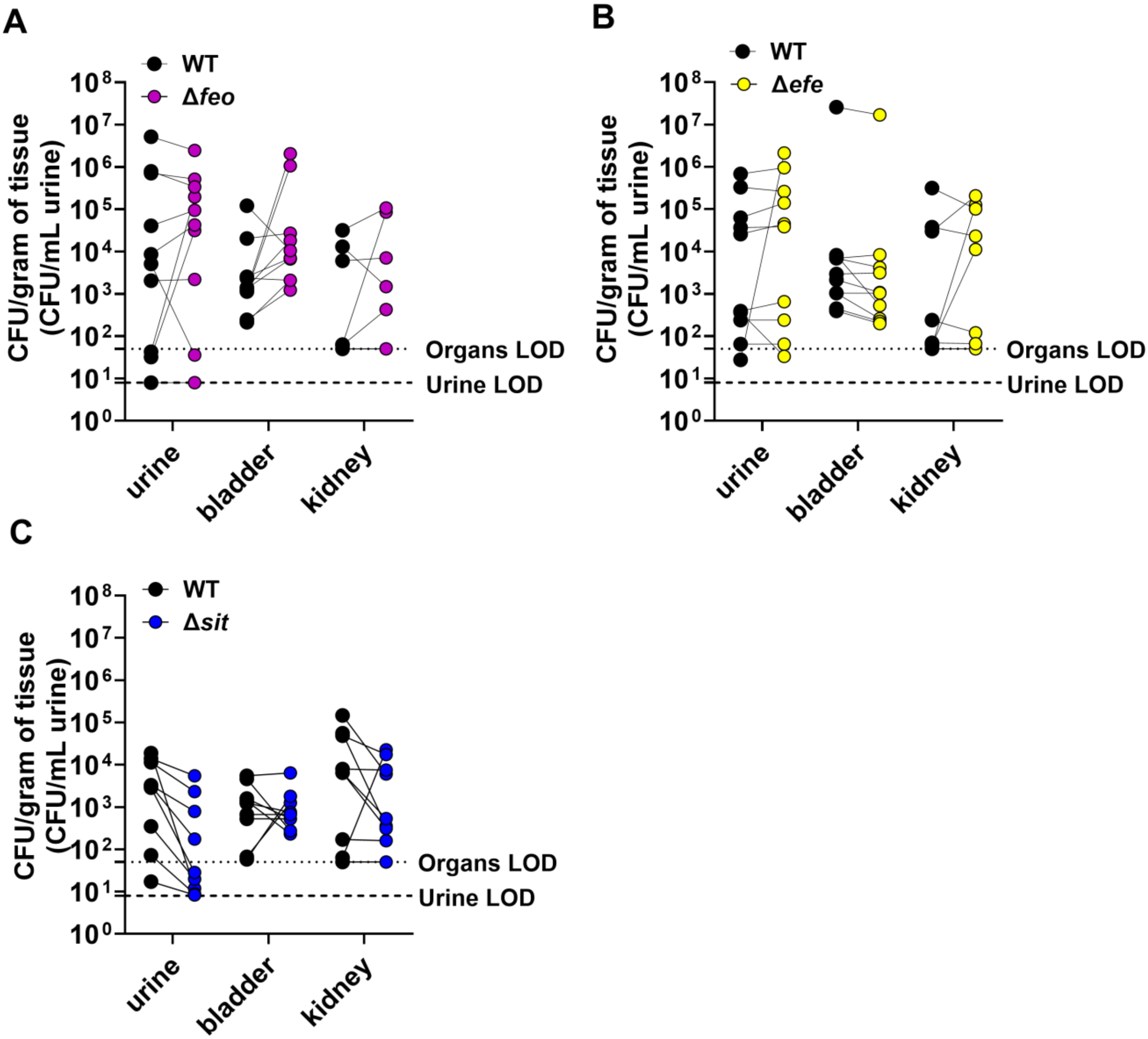

Figure S2. Mutant strains exhibit reduced colonization in mouse organs during co-infection with wild-type CFT073. Colony-forming units per milliliter (CFU/mL) were quantified from organ homogenates following co-infection of CBA/J mice with CFT073 wild type (WT) and individual mutant strains. Mice were transurethrally co-infected with a 1:1 ratio of WT and mutant bacteria. Bacterial burdens were assessed at 48 hours post-infection from plated urine, bladder, and kidney homogenates, both in the presence and absence of antibiotics. (**A**) WT vs. Δ*feo* (n=10), (**B**) WT vs. Δ*efe* (n=10), and (**C**) WT vs. Δ*sit* (n=9). Reduced CFU counts for the mutant strains relative to WT indicate a fitness disadvantage *in vivo* during urinary tract infection.

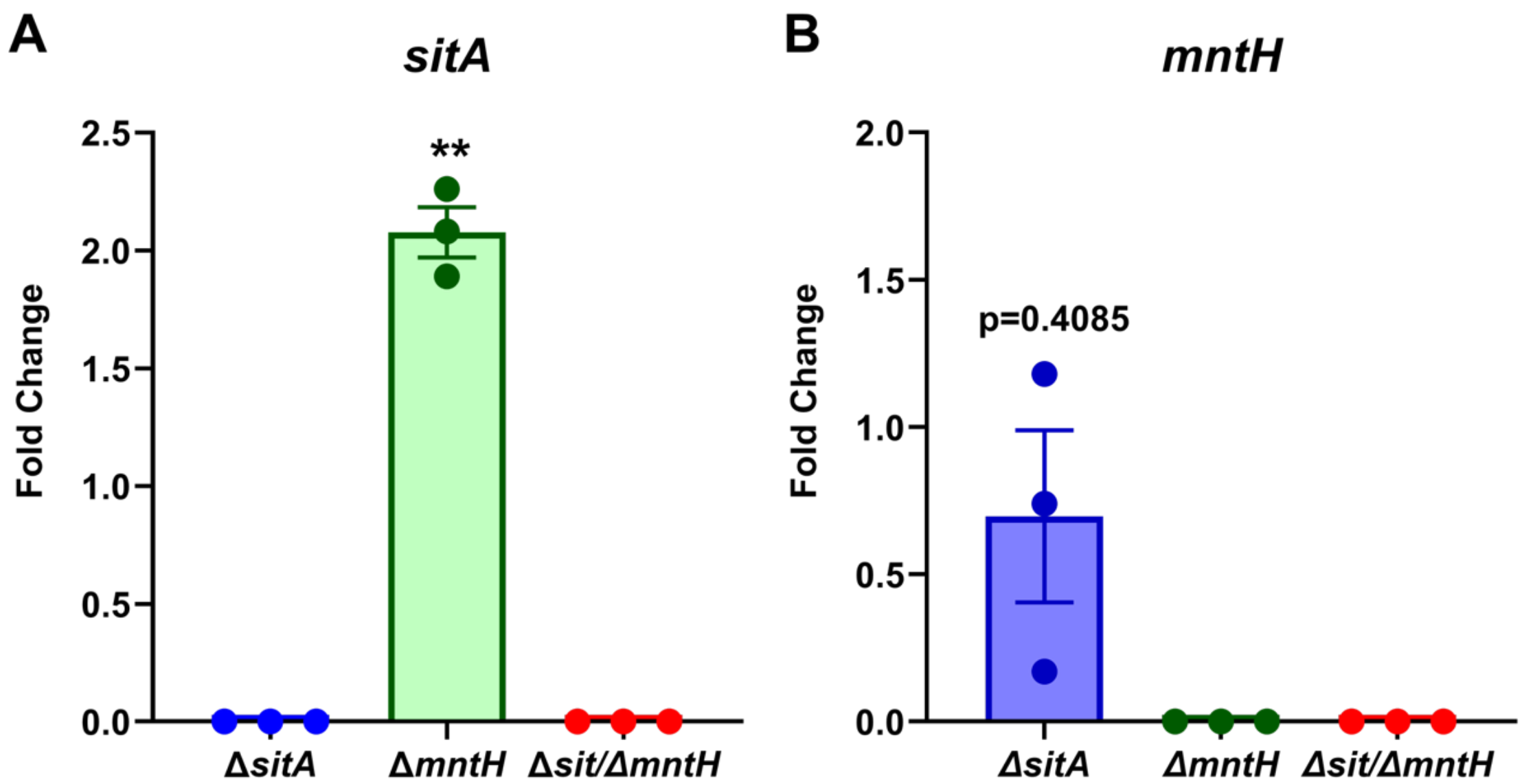

Figure S3. *sitA* is upregulated in Δ*mntH* mutant. Relative gene expression of (**A**) *sitA* and (**B**) *mntH* was measured in Δ*sit*, Δ*mntH*, and Δ*sit*/Δ*mntH* mutants in comparison to WT using qRT-PCR and *gapA* as the reference gene. All strains were grown in MOPS medium supplemented with 0.2% glucose. Bars represent the mean of three biological replicates; individual data points are shown as dots. Statistical significance was assessed using one sample t-test. (**, $P < 0.01$).

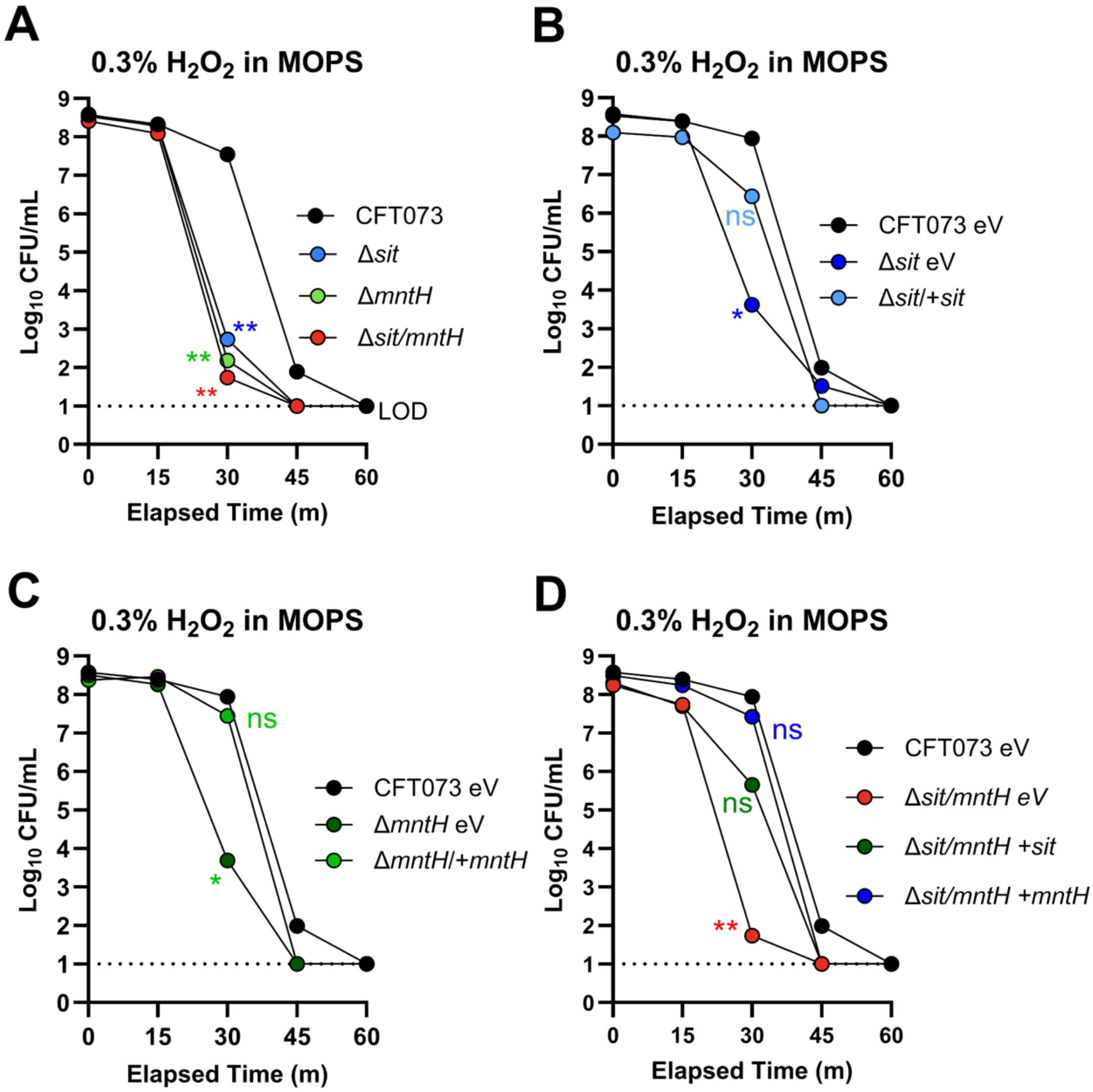

Figure S4. Δ*sit*, Δ*mntH*, and Δ*sit*Δ*mntH* mutants exhibit increased sensitivity to oxidative stress. (**A**) Wild-type CFT073 and mutant strains (Δ*sit*, Δ*mntH*, and Δ*sit*/Δ*mntH*) were exposed to oxidative stress by treatment with 0.3% hydrogen peroxide in MOPS minimal medium. All three mutants showed increased sensitivity compared to the wild type (WT). (**B-D**) Complementation of the deleted genes demonstrated varying levels of restored survival when compared to the WT. (**B**) Partial restoration in the Δ*sit* strain complemented with *sit*, (**C**) Full restoration in the Δ*mntH* strain complemented with *mntH*, and (**D**) Full restoration in the Δ*sit*Δ*mntH* strain complemented with *mntH*, all relative to WT. Empty vector (eV) plasmids were introduced into WT and mutant strains to control for any off-target effects of the plasmid backbone. Each strain was inoculated at approximately 10^8 CFU/mL, and viable colony counts were determined at 15-minute intervals post-inoculation by serial dilution plating. Data represent the median of three biological replicates. Statistical significance was assessed using one-way ANOVA (*$P < 0.05$; **$P < 0.01$).

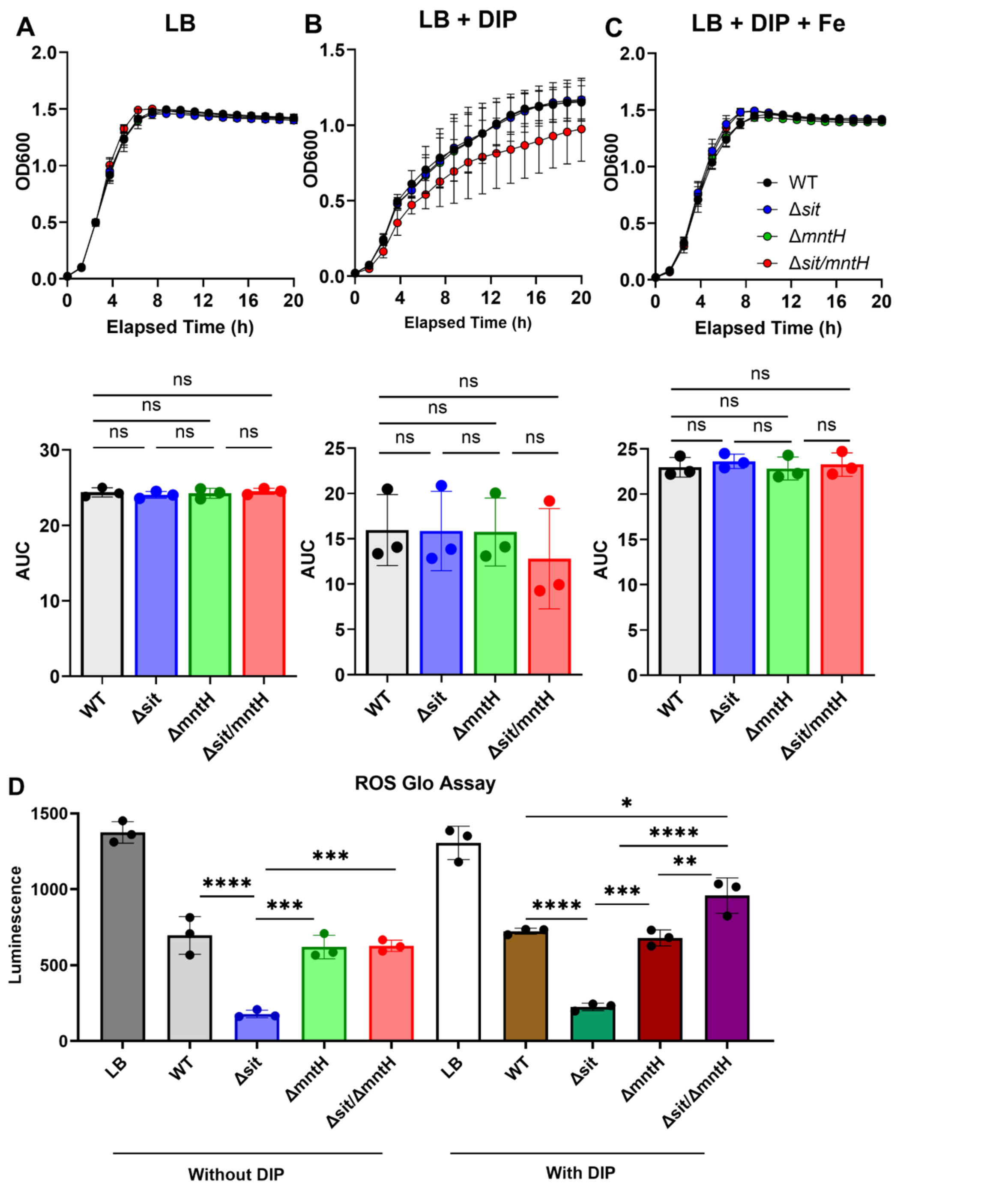

Figure S5. Altering iron levels in LB medium does not significantly affect growth of transport mutants Δ*sit*, Δ*mntH*, and Δ*sit*/Δ*mntH*. Bacterial growth of WT CFT073 and mutants Δ*sit*, Δ*mntH*, and Δ*sit*/Δ*mntH* was measured in (**A**) LB medium. We then added (**B**) 150 μM of iron-specific chelator 2,2-dipyridyl (DIP) to LB medium to create an iron-depleted condition. (**C**) LB and DIP supplemented with 36 μM $FeCl_3$ comprised the iron-replete condition. OD600 was taken over the course of 20 hours. Statistical significance was assessed for panel A-C using ordinary one-way ANOVA with Tukey's multiple comparisons test (ns means non-significant). (**D**) ROS-glo assay; a luminescence-based assay was used to measure the relative H_2O_2 concentration in media (LB) with and without iron chelator DIP. Data shown are the averages from three to five biological replicates, and error bars represent SEM. Statistical significance was assessed using ordinary one-way ANOVA with Tukey's multiple comparisons test. Comparisons without marked significance were determined to show no significant difference. ns, non-significant, *, $P < 0.05$, **, $P < 0.01$, ***, $P < 0.001$, ****, $P < 0.0001$.

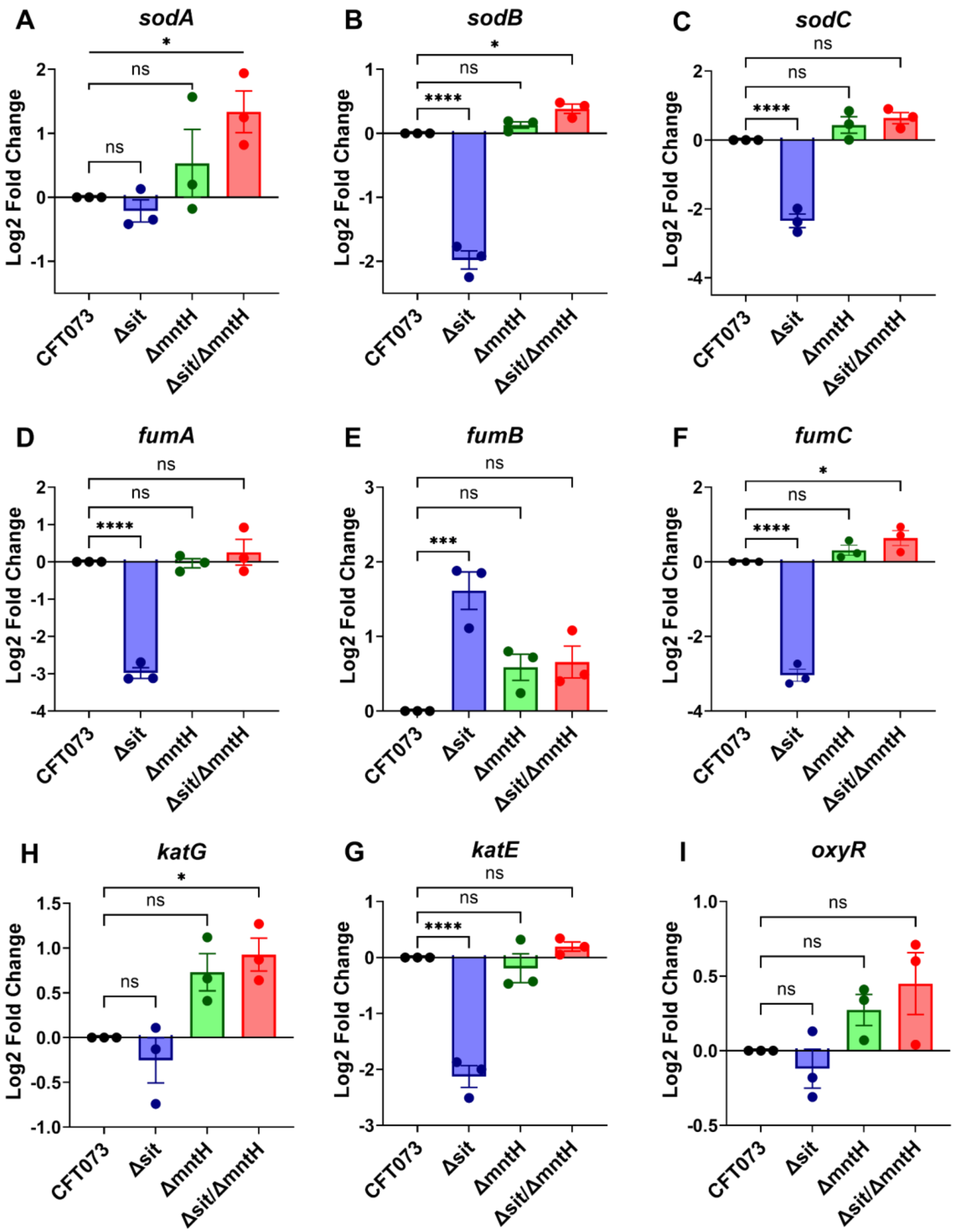

Figure S6. The *sitABCD* mutant exhibits metabolic reprogramming compared to WT. Relative expression of (**A**) *sodA*, (**B**) *sodB*, (**C**) *sodC*, (**D**) *fumA*, (**E**) *fumB*, (**F**) *fumC*, (**G**) *katG*, (**H**) *katE*, and (**I**) *oxyR* were measured in Δ*sit*, Δ*mntH*, and Δ*sit/*Δ*mntH* strains compared to WT by qRT-PCR using *gapA* as the reference gene. All strains were grown in LB medium for 3 hours. Bars represent the mean of three biological replicates, with individual data points shown as dots. Statistical significance was determined using one-way ANOVA followed by Dunnett's multiple-comparison test (*, $P < 0.05$; **, $P < 0.01$; ***, $P < 0.001$; ****, $P < 0.0001$).

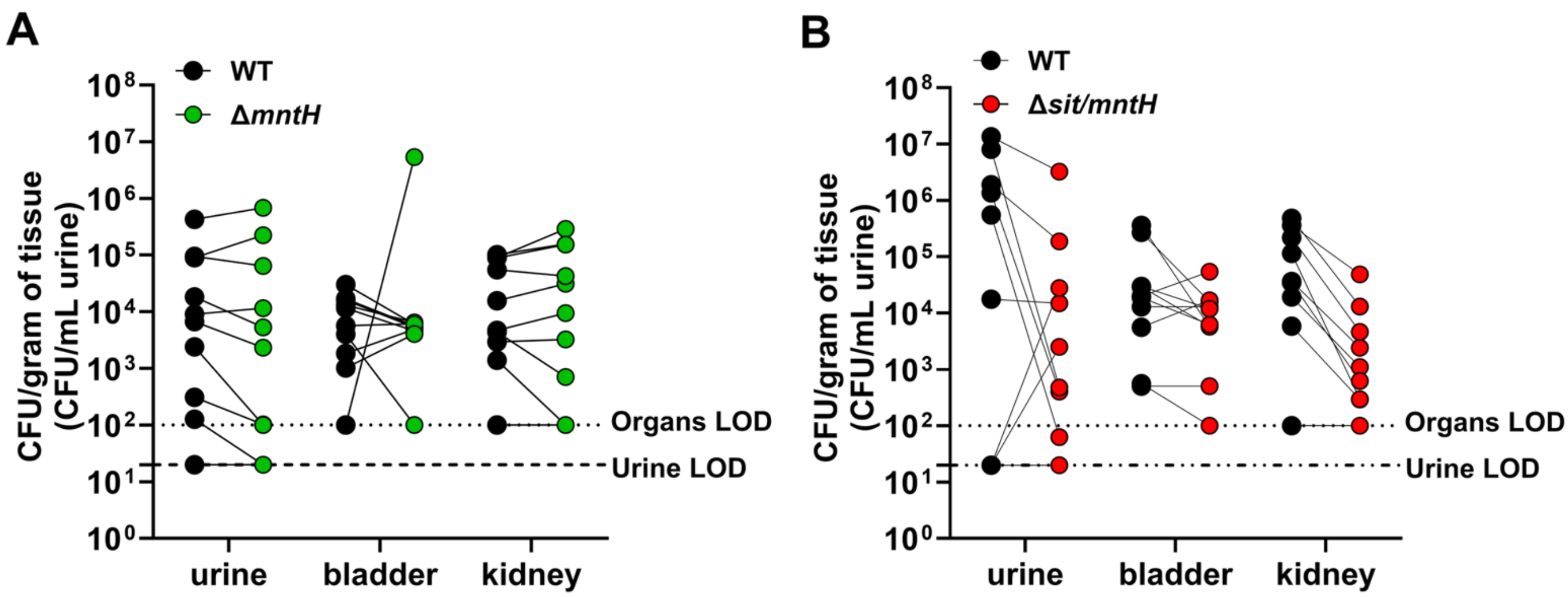

Figure S7. Mutant strains exhibit reduced colonization in mouse organs during co-infection with wild-type CFT073. Colony-forming units per milliliter (CFU/mL) were quantified from organ homogenates following co-infection of CBA/J mice with CFT073 WT and individual mutant strains. Mice were transurethrally co-infected with a 1:1 ratio of WT and mutant bacteria. Bacterial burdens were assessed at 48 hours post-infection from plated urine, bladder, and kidney homogenates, both in the presence and absence of antibiotics. (**A**) WT vs. Δ*mntH* (n=10), (**B**) WT vs. Δ*sit/ΔmntH* (n=10). Reduced CFU counts for the mutant strains relative to WT indicate a fitness disadvantage *in vivo* during urinary tract infection.
