# Supplementary material for: Importance of manganese uptake in uropathogenic Escherichia coli CFT073 during urinary tract infection
Source: mBio. 2026 Jun 26;17(8):e03395-25. doi: 10.1128/mbio.03395-25 (PMC13463694; doi:10.1128/mbio.03395-25)
Supplement: Supplementary file 2 [file mbio.03395-25-s0002.docx]

**SUPPLEMENTAL MATERIALS & METHODS**

**Bacterial strains and growth conditions.** UPEC strain CFT073 was isolated from the blood and urine of a patient with acute pyelonephritis (1). Bacteria were routinely cultured in lysogeny broth (LB) made up of 0.5g NaCl, 5g yeast extract, and 10g tryptone per liter at 37°C under aerated conditions and were inoculated from single colonies (mutants were grown in antibiotics). Overnight LB bacterial cultures were washed once with phosphate-buffered saline (PBS) and then inoculated 1:100 into 3 mL LB or filter-sterilized pooled human urine collected from at least four healthy female donors. Cultures were incubated with aeration at 37°C in a Bioscreen-C automated growth curve machine (Growth Curves USA), with a collection of optical density at 600 nm readings every 15 min for 20-24 hours. For *in vitro* iron-limitation experiments, wild-type (WT) and mutants were initially cultured with aeration at 37°C overnight in potassium morpholinopropane sulfonate (MOPS) containing 0.2% glucose, a medium that allows for optimal growth of *E. coli* (2). The following day they were diluted 1:100 in MOPS medium, or MOPS medium that had been treated with chelex-100, which removes metallic cations. Elements were then added back to chelexed MOPS individually; either 36µM FeCl_3_ or 50µM MnSO_4_. Growth was monitored using the Bioscreen-C automated growth curve machine as described above. For liquid CAS experiments, strains were grown in M9 medium supplemented with 0.4% glucose.

**Construction of Mutants and Complementation.** UPEC strain CFT073 mutants were generated using the lambda red recombinase system (3). In brief, using the primers from **Table 2** and plasmids from **Table 1,** *feoABC* and *mntH* genes were replaced with a chloramphenicol-resistance cassette from the pKD3 plasmid, *sitABCD* was replaced with a kanamycin-resistance cassette from the pKD4 plasmid, and *efeUOB* was replaced with a spectinomycin-resistance cassette from the pKD5 plasmid. Complementation of genes was achieved using the pGEN empty vector (eV) backbone, a low-copy-number, promoterless plasmid (4).

**Murine model of ascending UTI co-challenge experiments.** The individual fitness of each mutant was assessed in the CBA/J mouse model of ascending UTI (5). WT CFT073 and mutant strains were cultured overnight in LB broth under 37°C aerated conditions from a single colony. A bacterial suspension for inoculation was created with a 1:1 ratio of WT CFT073 to chloramphenicol-resistant mutants (f*eo* and *mntH*), kanamycin-resistant mutant (*sit*), or spectinomycin-resistant mutant (*efe*) in PBS. CBA/J female mice were 6 to 8 weeks old and 20 to 22 g (Jackson Laboratories) and were anesthetized with ketamine/xylazine and then infected transurethrally with 50 μL containing 2 × 10^8^ CFU per mouse using a sterile polyethylene catheter (0.28 mm x 0.61 mm) connected to an infusion pump (Harvard Apparatus). Input CFU/mL was confirmed by plating spiral dilutions (Spiral Biotech) onto LB agar plates with and without antibiotics. Bladders and kidneys were aseptically removed for each mouse, weighed, and then homogenized (OMNI International) in 3 mL of PBS. The homogenates were diluted and spiral-plated onto LB agar plates with and without antibiotics to determine the CFU output/g tissue for each strain per mouse. Counts were determined using QCount software (Spiral Biotech), and then the number of mutant CFU (antibiotic plates) was subtracted from the total number of CFU (plain plates) to determine the number of bacteria present in the input and output.

**CAS Plate Assay.** To assess siderophore production, the CAS agar assay was performed as previously described (6, 7). Bacterial strains were cultured overnight at 37^o^C in LB. Then, 5 μL of each overnight culture was spotted onto CAS agar plates and incubated at 37^o^C for 18-20 hours to allow for siderophore production and detection. A visible color change of agar from blue to orange around bacterial growth indicates siderophore production. The diameter (mm) of the zones was measured using a ruler and recorded.

**Hydrogen peroxide assays.** Overnight cultures of ∆*sit*, ∆*mntH*, and ∆*sit/mntH* were inoculated into LB medium overnight. The next day, cultures were normalized to approximately 108 CFU/mL, then washed in MOPS with an iron-free formulation of nutrient mix and 0.2% glucose. All hydrogen peroxide assays were performed under static conditions at room temperature, and samples were collected at 15, 30, 45, and 60 minutes post-inoculation. Following incubation of LB agar plates at 37ºC for 18h, viable colonies were enumerated at countable dilutions.

**ROS glo Assay.** Bacterial strains were grown overnight at 37^o^C with aeration The next day, bacteria were grown in either LB only or LB + [300 µM] iron chelator 2,2 dipyridyl (DIP). 30µl of overnight culture was added. Blank LB with and without DIP were used as controls. The cultures were incubated for 3 hours at 37^o^C with aeration. After 3 hours of incubation, 100µl from each culture was spun down at 10,000 x g for 3 minutes. 80µl of the supernatant was transferred to 96 well plates while the pellet was resuspended in a fresh 100µl LB only and LB + DIP. To each of the samples, 20µl of H_2_O_2_ substrate solution (per ROS-glo kit: 200µl buffer + 2.5µl substrate) was added. These were incubated for 5 minutes, afterwards, Luciferin Detection Reagent (LDR) solution was added according to the instructions on the kit. Following this, luminescence was measured on a [BioTek] plate reader at 2 hours.

**Liquid CAS Assay.** Strains were cultured overnight in LB with aeration at 37°C. The overnight cultures were harvested by centrifugation, washed with PBS, diluted 1:100 into M9 minimal medium supplemented with 0.4% glucose, and incubated overnight, with aeration at 37°C. Cultures were pelleted by centrifugation, and 100 μL of supernatants was combined with 100 μL of chrome azurol S (CAS) shuttle solution prepared according to an established protocol (8) with 10 mM EDTA used a positive control (AmBeed catalog no.A354454-100g). Solutions were incubated at room temperature for 30 minutes and the absorbance at 630 nm was recorded.

**REFERENCES**

1.	Mobley HL, Green DM, Trifillis AL, Johnson DE, Chippendale GR, Lockatell C V., et al. Pyelonephritogenic *Escherichia coli* and killing of cultured human renal proximal tubular epithelial cells: role of hemolysin in some strains. Infect Immun [Internet]. 1990 [cited 2025 Feb 20];58(5):1281–9. Available from: https://pubmed.ncbi.nlm.nih.gov/2182540/

2.	Neidhardt FC, Bloch PL, Smith DF. Culture medium for enterobacteria. J Bacteriol [Internet]. 1974 [cited 2025 Feb 20];119(3):736–47. Available from: https://pubmed.ncbi.nlm.nih.gov/4604283/

3.	Datsenko KA, Wanner BL. One-step inactivation of chromosomal genes in *Escherichia coli* K-12 using PCR products. Proc Natl Acad Sci U S A [Internet]. 2000 Jun 6 [cited 2025 Feb 20];97(12):6640–5. Available from: https://pubmed.ncbi.nlm.nih.gov/10829079/

4.	Lane MC, Alteri CJ, Smith SN, Mobley HLT. Expression of flagella is coincident with uropathogenic *Escherichia coli* ascension to the upper urinary tract. Proc Natl Acad Sci U S A [Internet]. 2007 Oct 16 [cited 2025 Feb 20];104(42):16669–74. Available from: https://pubmed.ncbi.nlm.nih.gov/17925449/

5.	Shea AE, Marzoa J, Himpsl SD, Smith SN, Zhao L, Tran L, et al. *Escherichia coli* CFT073 Fitness Factors during Urinary Tract Infection: Identification Using an Ordered Transposon Library. Appl Environ Microbiol [Internet]. 2020 Jul 1 [cited 2025 Feb 20];86(13). Available from: https://pubmed.ncbi.nlm.nih.gov/32358013/

6.	Schwyn B, Neilands JB. Universal chemical assay for the detection and determination of siderophores. Anal Biochem [Internet]. 1987 [cited 2025 Feb 20];160(1):47–56. Available from: https://pubmed.ncbi.nlm.nih.gov/2952030/

7.	Himpsl SD, Pearson MM, Arewng CJ, Nusca TD, Sherman DH, Mobley HLT. Proteobactin and a yersiniabactin-related siderophore mediate iron acquisition in *Proteus mirabilis*. Mol Microbiol [Internet]. 2010 Oct [cited 2025 Feb 20];78(1):138–57. Available from: https://pubmed.ncbi.nlm.nih.gov/20923418/

8.	Himpsl SD, Mobley HLT. Siderophore Detection Using Chrome Azurol S and Cross-Feeding Assays. Methods Mol Biol [Internet]. 2019 [cited 2025 Oct 28];2021:97–108. Available from: https://pubmed.ncbi.nlm.nih.gov/31309499/
